# Skin-interfaced multimodal sensing and tactile feedback system as enhanced human-machine interface for closed-loop drone control

**DOI:** 10.1126/sciadv.adt6041

**Published:** 2025-03-26

**Authors:** Chunki Yiu, Yiming Liu, Wooyoung Park, Jian Li, Xingcan Huang, Kuanming Yao, Yuyu Gao, Guangyao Zhao, Hongwei Chu, Jingkun Zhou, Dengfeng Li, Hu Li, Binbin Zhang, Lung Chow, Ya Huang, Qingsong Xu, Xinge Yu

**Affiliations:** ^1^ Department of Biomedical Engineering, City University of Hong Kong, Hong Kong, China.; ^2^ Hong Kong Center for Cerebra-Cardiovascular Health Engineering, Hong Kong Science Park, New Territories, Hong Kong, China.; ^3^ Department of Electrical Engineering and Information Systems, The University of Tokyo, Tokyo, Japan.; ^4^ Department of Electromechanical Engineering, Faculty of Science and Technology, University of Macau, Taipa, Macau, China.; ^5^ Institute of Digital Medicine, City University of Hong Kong, Kowloon, Hong Kong, China.; ^6^ Hong Kong Institute for Clean Energy, City University of Hong Kong, Kowloon, Hong Kong, China.

## Abstract

Unmanned aerial vehicles have undergone substantial development and market growth recently. With research focusing on improving control strategies for better user experience, feedback systems, which are vital for operator awareness of surroundings and flight status, remain underdeveloped. Current bulky manipulators also hinder accuracy and usability. Here, we present an enhanced human-machine interface based on skin-integrated multimodal sensing and feedback devices for closed-loop drone control. This system captures hand gestures for intuitive, rapid, and precise control. An integrated tactile actuator array translates the drone’s posture into two-dimensional tactile information, enhancing the operator’s perception of the flight situation. Integrated obstacle detection and neuromuscular electrical stimulation–based force feedback system enable collision avoidance and flight path correction. This closed-loop system combines intuitive controls and multimodal feedback to reduce training time and cognitive load while improving flight stability, environmental awareness, and the drone’s posture. The use of stretchable electronics also addresses wearability and bulkiness issues in traditional systems, advancing human-machine interface design.

## Teaser

A multimodal human-machine interface has been developed to aid the control and perception capabilities of the drone operator.

## INTRODUCTION

Unmanned aerial vehicles, commonly referred to as “drones,” have garnered considerable attention and substantial market expansion in recent years (*1*, *2*). The number of registered commercial drones reached 1.78 million in 2023 and is projected to surge to 1.88 million by 2028 in the United States alone (*1*), and the global drone market is projected to reach €53.1 billion by 2025 (*3*). An increasing number of drones are being used in various facets of people’s daily lives, encompassing domains such as transportation, infrastructure, and other sectors (*3*). Substantial improvements have been achieved for autonomous drones by merging artificial intelligence and control algorithms in recent years, while interaction between drones and users is still far behind, which is the key reason restricting drone navigation in complex and rapidly changing environments (*4*–*6*). In many situations, human is the unreplaceable operator, who demonstrates better robustness in unexpected and dynamic environments (*7*, *8*). So, developing good control technologies, from software to hardware, is equally important. Presently, the predominant control methods for drones involve joystick-based controllers or touch interfaces, such as smartphones, which encounter challenges related to increased control complexity, prolonged training periods, and higher cognitive demand (*9*–*13*). In response to these challenges, an increasing number of research works have been done on various intuitive control methods for drone operation. These methods include brain-computer interface systems based on electroencephalography (EEG) (*14*–*17*), speech control (*18*–*20*), gesture control based on electromyography (EMG) (*21*–*24*), computer vision gesture classification (*9*, *11*, *25*–*27*), gesture recognition using mechanical sensors or inertial measurement units (IMUs) (*12*, *28*–*30*), and gesture-tapping command input facilitated by innovative tactile interfaces (*31*). Some studies have also integrated multiple control methods to enhance overall performance (*32*, *33*). These intuitive control approaches have demonstrated the ability to reduce operator training time due to their high learnability, minimizing cognitive load and allowing users to focus on flight tasks. Moreover, they offer a heightened sense of control and driving satisfaction (*12*, *13*).

Unfortunately, the development of strong closed-loop interaction between the user and the drone, associated with rich information sharing between the drone and the user, is still in a very infant stage, restricting the better operation of the drone. Throughout the evolution of closed-loop human-machine interaction design, continuous feedback and interaction from the machine to the operator have remained vital for managing challenging situations, as system errors are inevitable (*34*). In addition to basic visual feedback, research in conventional manned aircraft control systems suggests that providing feedback in additional dimensions, such as haptic and kinesthetic, to form a multimodal feedback system, means a lot to enhance control performance and facilitate accurate control decision-making (*35*–*37*). Currently, most of the commercially available drones offer only visual feedback from a first-person perspective (FPV). However, the limited range of vision may not provide adequate sensory information and could lead to delayed responses to changes in the flying environment (*10*). Furthermore, certain critical sensory perceptions, such as those provided by the human vestibular system, are essential for manned aircraft pilots to be aware of the aircraft’s posture and motion. These sensory inputs are absent in drones due to their unmanned nature (*38*, *39*). Some research aims to integrate tactile/cutaneous feedback, which provides a sensation of “touch” (*4*, *40*, *41*), or force feedback, which directly applies force or imparts a kinesthetic sensation on the user’s body (*42*–*45*), into drone control programs to enhance control efficiency. These feedback systems still provide very limited improvement in closed-loop interaction, mainly because of dull function and low resolution. Therefore, how to realize the multimodal sensing and feedback system that can be closely integrated with users is the key for closed-loop drone control with strong human-machine interaction.

In this study, we report an enhanced human-machine interface based on skin-integrated multimodal sensing and feedback devices for closed-loop drone control. The system incorporates an IMU to capture user hand movements and correlate them with drone control commands, enabling intuitive drone control and meeting the demand for fast and precise responses to user gestures. A multimodal feedback system comprising fundamental FPV visual feedback, tactile feedback, and electrical stimulation is developed to provide rich information to users. Distinguished from a conventional system with only FPV visual information, the tactile feedback adopting miniaturized vibration actuators can mimic and transmit the drone’s “sensations” to users, while the electrical stimulation based on neuromuscular electrical stimulation (NMES) technique can generate strong force to allow the user to subconsciously correct flight path and avoid collision. The adoption of thin, soft, flexible, and stretchable electronic design endows the system with a high degree of wearability and portability. Moreover, it circumvents issues of bulkiness while integrating additional functionality with enhanced performance compared to previous attempts at human-drone interface development (Table 1). The reported technology offers a straightforward means of drone control reducing training periods and cognitive demand, augmenting drone flight stability by enabling awareness of the surrounding flying environment and the posture of the drone.

**Table 1.** Comparative analysis between our design and existing human-drone interface systems.

Authors	Year	Control method	Feedback method	Wearable/portable	Stretchable electronic design
Lee *et al.* (*16*)	2021	EEG-based drone control	–	Yes	No
Contreras *et al.* (*18*)	2020	Speech recognition	–	Yes	No
Sanchez *et al.* (*22*)	2017	EMG-based drone control	–	Yes	No
Jeong *et al.* (*23*)	2013	EMG-based drone control	–	Yes	Yes
Kwon *et al.* (*24*)	2020	EMG-based drone control	–	Yes	Yes
Khaksar *et al.* (*26*)	2023	Gesture (computer vision–based)	–	Yes	No
Cherpillod *et al.* (*28*)	2019	Gesture (mechanical sensor)	Visual feedback, haptic feedback, vestibular feedback	No	No
Choi *et al.* (*29*)	2019	Gesture (mechanical sensor)	–	Yes	Yes
Li *et al.* (*30*)	2023	Gesture (mechanical sensor)	–	Yes	Yes
Xiang *et al.* (*32*)	2022	Gesture (computer vision–based) + speech recognition	Visual feedback	Yes	No
Fernández *et al.* (*33*)	2016	Gesture (computer vision–based) + speech recognition	Visual feedback	Yes	No
Macchini *et al.* (*4*)	2020	Gesture (IMU)	Tactile feedback (5 actuators)	Yes	No
Ruff *et al.* (*40*)	2000	Joystick controller	Visual feedback, force feedback	No	No
Tsykunov *et al.* (*41*)	2019	Gesture	Tactile feedback (5 actuators)	Yes	No
Goodrich *et al.* (*42*)	2011	Joystick controller	Visual feedback, force feedback (haptic stick)	No	No
Rognon *et al.* (*12*)	2018	Gesture (IMU)	Visual feedback, force feedback (exoskeleton)	Yes	No
Lee and Yu (*70*)	2023	Gesture (IMU)	Tactile feedback (1 actuator)	Yes	No
Our design	–	Gesture (IMU)	Visual feedback, tactile feedback (3-by-3 array), force feedback (NMES)	Yes	Yes

## RESULTS

**Fig. 1.**
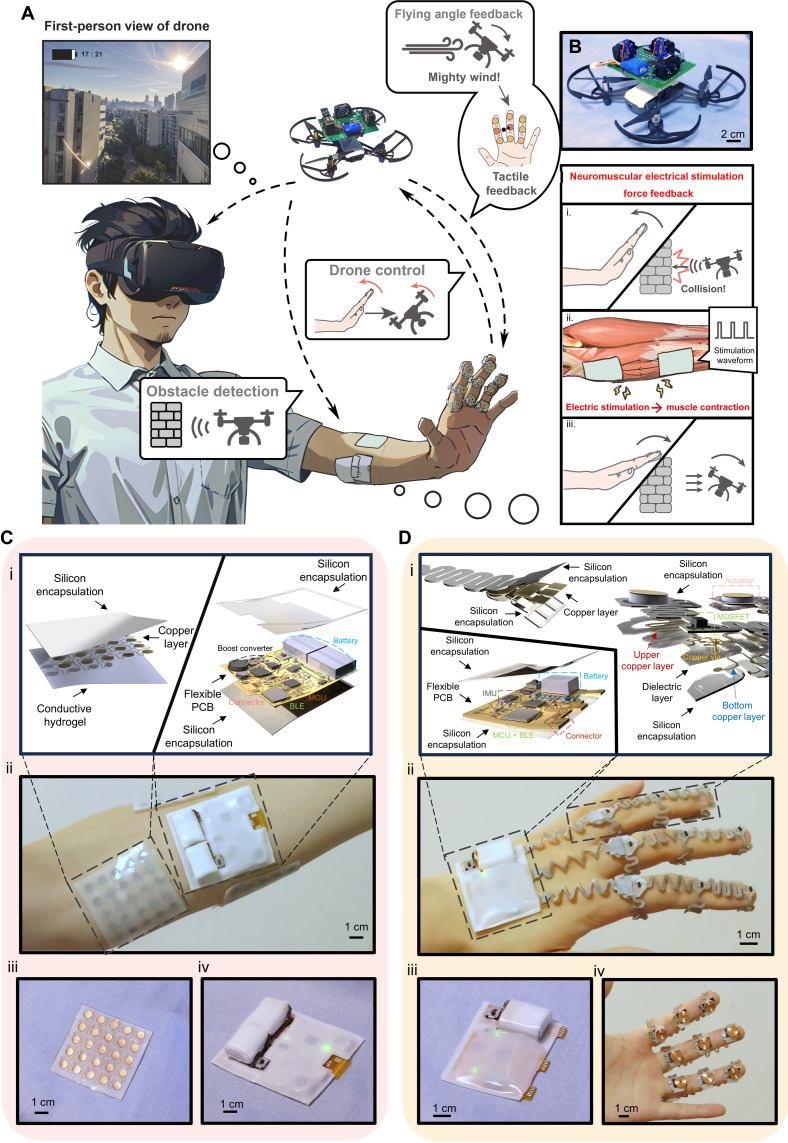
Concept illustration and photos of the multimodal closed-loop system. (**A**) Conceptual illustration demonstrating various functionalities of the system. (**B**) Drone equipped with the obstacle detection system, shown in an optical view. (**C**) (i) 3D structural depiction of the NMESF electrode and control units. (ii) Optical view of the NMESF module attached to the user’s forearm. (iii) Optical view of the stimulation electrode. (iv) Optical view of the NMESF control unit. (**D**) (i) 3D structural representation of the DCTF control unit, connector, and tactile feedback array. (ii) Optical view of the DCTF module mounted on the user’s hand. (iii) Optical view of the DCTF control unit. (iv) Optical view of the actuator array with nine actuators.

The conceptual depiction of the closed-loop system is shown in Fig. 1A. The system integrates four primary functionalities: FPV visual feedback provided by the drone’s system, intuitive drone control through hand gestures, vibration feedback mimicking human vestibular system, and a strong force feedback module. The FPV provides visual information of the drone based on the built-in camera. The drone control associates with the gesture command decoded by motion detection with an IMU. The vibration feedback is based on an electromagnetic-driven method to offer real-time flight angle or posture information, and thus allows dynamic monitoring of the flying and aerodynamic conditions surrounding the drone. The force feedback system is based on the NMES technique, which is collaborated with the obstacle detection system. This system delivers electrical stimulation to the user’s muscles, resulting in muscle contraction to generate tension and force as a form of force feedback. The force feedback mechanism will counteract or reverse hand motion upon detection of obstacles in the corresponding direction, aimed at mitigating the risk of potential collisions and accidents.

**Fig. 2.**
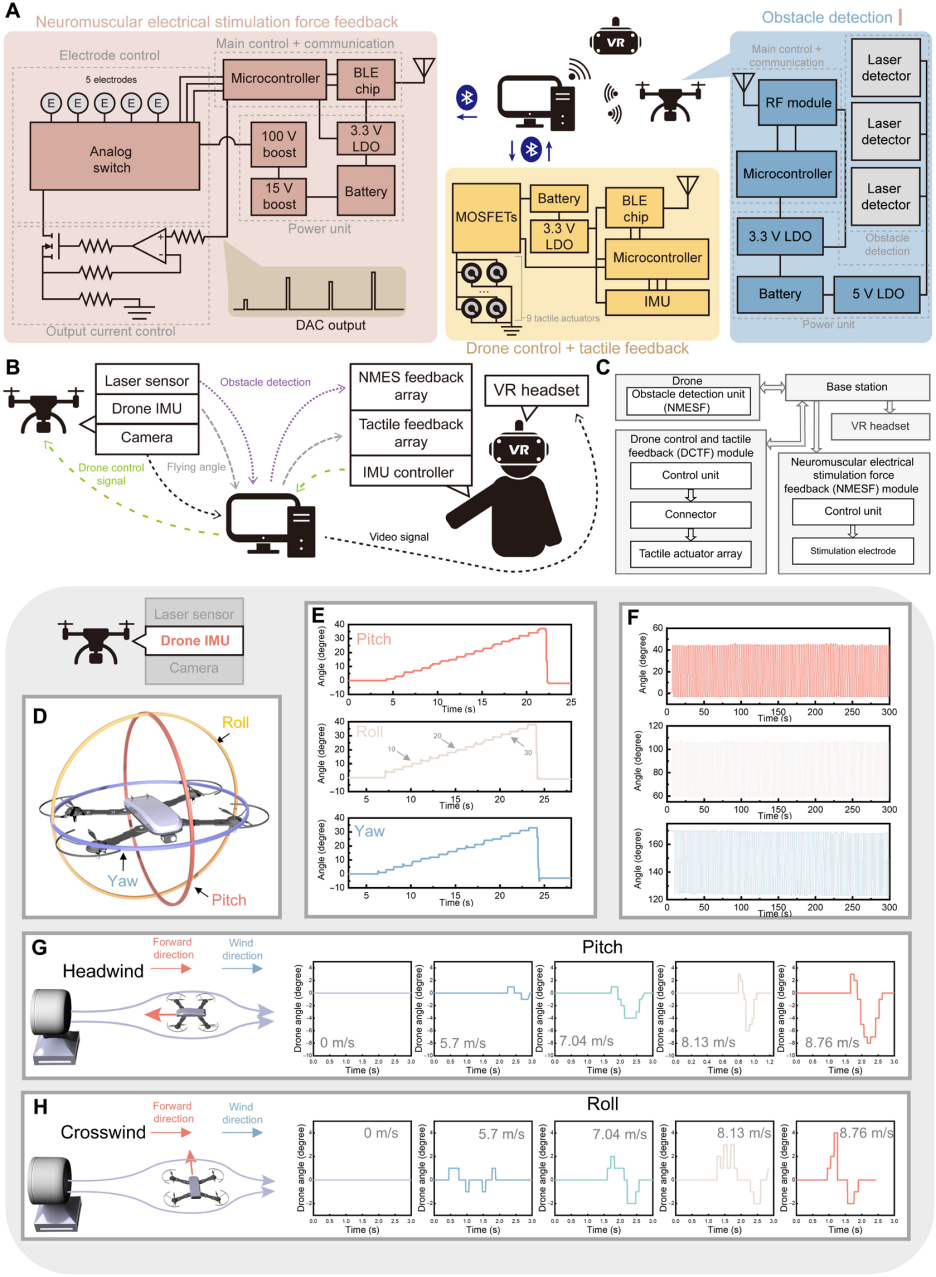
Hardware structure, working logic, and performance of IMU on the drone. (**A**) Block diagram illustrating the three primary hardware modules within the system, along with the functional units under each system. DAC, digital-to-analog; LDO, low-dropout regulator. (**B**) Depiction of the operational logic and workflow of the system. (**C**) Block diagram illustrating the hardware architecture of the system. (**D**) Three rotation axes of the drone. (**E**) Assessment of the accuracy of the drone’s IMU across all three axes. (**F**) Evaluation of the long-term stability of the drone’s IMU. (**G**) Relationship between the pitch rotation of the drone and the headwind at various wind speeds. (**H**) Relationship between the roll rotation of the drone and the crosswind at various wind speeds.

The vibration feedback and intuitive control are managed by the drone control and tactile feedback (DCTF) module (Figs. 1D and 2A and fig. S1). The DCTF module consists of two units: the control unit and the tactile actuator array. The DCTF control unit [Fig. 1D(iii) and fig. S6A], constructed from a flexible printed circuit board (FPCB) and encased in a silicone film, is affixed to the dorsum of the user’s hand. The DCTF incorporates an IMU on the circuit to monitor variations in the user’s hand angle. Orientation data are gathered by a microcontroller (MCU) and transmitted wirelessly via a Bluetooth Low Energy (BLE) chip. In addition, the MCU converts tactile feedback commands received from the BLE chip into digital output signals, which are then conveyed to the tactile actuator array via a connector. The tactile actuator array and the connector [Fig. 1D(iv) and figs. S1 and S3A] are equipped with metal oxide semiconductor field-effect transistors (MOSFETs) and vibrational tactile actuators (*46*), constituting a 3-by-3 actuator array capable of generating two-dimensional (2D) tactile feedback on the user’s finger, which exhibits the highest sensitivity to tactile stimuli compared to all other body parts (*47*, *48*). Both the connector and the tactile actuator array use a thin (500 μm) and stretchable electronic design, featuring a well-established serpentine structure (*49*–*51*) that allows good stretchability up to 50% stretching (fig. S3, B to E). This design both accommodates the flexion of the human finger and allows the device to be positioned close to the user’s skin, enhancing comfort and user experience. The tactile actuator array adopts a multilayer design aimed at reducing circuit dimensions and mitigating circuit wiring complexities [Fig. 1D(i) and fig. S2]. This design comprises two layers of copper circuitry attached and separated by polydimethylsiloxane (PDMS) substrate and dielectric layer, respectively. The connection between the two copper layers is established through vias and copper pins with a diameter of 0.4 mm. Furthermore, the upper copper layer is encapsulated by PDMS, leaving only the soldering pad exposed. This arrangement facilitates the soldering of the integrated chip while ensuring the attachment of the copper layer during device deformation. Despite comprising only a single copper layer, the connector also incorporates PDMS substrate and encapsulation to ensure its mechanical performance.

Another important functional module within the system is the NMES force feedback (NMESF) module (Figs. 1C and 2A). This module is responsible for controlling the generation of electrical stimulation across multiple muscle groups to provide force feedback. The units comprising the NMESF module can also be categorized into three units: the control unit [Fig. 1C(iv) and fig. S6B], the stimulation electrode [Fig. 1C(iii)], and the obstacle detection unit (Fig. 1B and fig. S6C). The obstacle detection unit is positioned atop the drone, with three laser detectors detecting obstacles to the left, right, and rear of the drone (areas not covered by the drone camera). The obstacle information is wirelessly transmitted via a radio frequency (RF) module to the drone’s base station, where the data are subsequently processed and distributed to other modules. Conversely, the NMESF control module, which attached on the user’s forearm, will receive stimulation commands from the base station. The MCU, analog switch, and current control circuit on the control unit are coordinated to deliver a specific amount of current to the targeted muscle. A two-stage boost circuit provides sufficient voltage (>100 V) to overcome the high impedance of the human body and deliver the required current (Fig. 2A). The current is then applied to the muscle through the electrode. The electrode also adopts a stretchable substrate and serpentine copper design, allowing it to bend, flex, or stretch in various dimensions (fig. S5, A and B). In addition, a layer of stretchable and replaceable hydrogel is affixed to the electrode to reduce the electrode-to-skin impedance [Fig. 1C(i) and fig. S5C]. The hardware block diagram of the entire system is presented in Fig. 2C.

The operational logic flow of the system is delineated in Fig. 2B and fig. S8. System operation starts with the capture of hand orientation data by the IMU. The IMU data, gathered by the DCTF modules, are wirelessly transmitted to the base station, a computer. Subsequently, the base station converts the angle data into drone control commands and transmits them to the drone via a wireless local area network (WLAN), thereby directing the drone’s movement according to hand gestures. Conversely, the orientation data and the flying speed of the drone are transmitted back to the base station for the estimation of flying posture and aerodynamic conditions. These data are used to generate a 2D tactile feedback map, and the control commands for each actuator are transmitted back to the DCTF modules to induce tactile feedback on the user’s finger. This, coupled with intuitive control facilitated by hand gestures, constitutes the first control feedback loop. In addition to tactile feedback, the obstacle detection system mounted on the drone detects obstacle presence and distance from the drone. The data are transmitted to the base station via the RF module and translated into muscular stimulation commands. These commands are transmitted via BLE to the NMESF control unit, which generates electrical stimulation and force feedback for the user. As the drone is controlled by hand movements, the NMESF and intuitive control form the second feedback loop. Moreover, the integrated camera on the drone sends real-time video signals back to the base station via WLAN. The base station subsequently transfers the signals wirelessly to a virtual reality (VR) headset, forming the visual feedback loop. Together with intuitive control, tactile feedback, and force feedback, these loops comprise the multimodal closed-loop control system. Figure S9 illustrates how the system is mounted on the user’s body and the drone, and each component of the system will be elaborated in the following sections.

The performance of the drone’s IMU is assessed, with the 3D rotation planes—pitch, roll, and yaw—depicted in Fig. 2D. Real-time rotational information of the drone across these three planes is recorded during flight. The accuracy of the IMU is evaluated, and the results are shown in Fig. 2E. During testing, the drone undergoes rotation from 0° to 36° in 2° increments on a robotic arm. The angle readings from the drone for the pitch, roll, and yaw dimensions consistently align with the test settings, demonstrating the high accuracy of the IMU and its ability to capture variations in a drone angle during flight. The repeatability of the angle measurement with 2° variations has been shown in fig. S10. Furthermore, a long-term test is conducted wherein the robotic arm, affixed with the drone, is continuously rotated by 45° for 300 s. The test duration is limited to 300 s, as the drone system automatically shuts down after this period if no control command is issued. The results across all three dimensions (Fig. 2F) exhibit high repeatability, with a consistent 45° variation observed during the testing period. Only the yaw direction demonstrates a minor shift of 2° to 3°, attributed to the magnetic compass used in the nine-axis IMU, which is inherently susceptible to shifting or interference from the surrounding magnetic anomaly field when compared to pitch and roll axes (*52*, *53*). The high repeatability and stability, coupled with high accuracy, affirm that the drone’s integrated IMU can provide reliable angular data during flight.

Following the evaluation of the IMU performance, an examination of the relationship between drone angles during flight and the surrounding wind speed and direction is undertaken. Analogous to other aircraft, drone flight can be substantially influenced by aerodynamic conditions such as wind speed and direction. Operators may require immediate adjustments to correct flight posture and heading direction upon encountering strong winds. Detection of the aerodynamic situation is therefore important, considering that airflow is imperceptible in visual feedback. Introducing specific sensors is a way to monitor these aerodynamic factors. However, the incorporation of additional sensors, particularly when drones are subjected to multidirectional winds or turbulence, results in increased size and weight, imposing a notable burden on the drone. Conversely, wind can also affect flight posture due to air resistance, prompting an investigation into the correlation between unexpected variations in drone flight angle and sudden changes in external aerodynamic conditions. In Fig. 2 (G and H), the drone is subjected to headwind (wherein the wind flows from the forward direction of the drone) and crosswind (wherein the wind flows from the side of the drone) conditions, respectively. Analysis of the data reveals that, with increasing wind speed, the pitch and roll axes experience augmented variations in drone angle, with the biggest angle variation occurring at a wind speed of 8.76 m/s. This proportional relationship underscores that changes in drone angle during flight can reflect marked alterations in aerodynamic conditions. Consequently, drone angular data are transmitted back to the base station for aerodynamic situation estimation within our system.

**Fig. 3.**
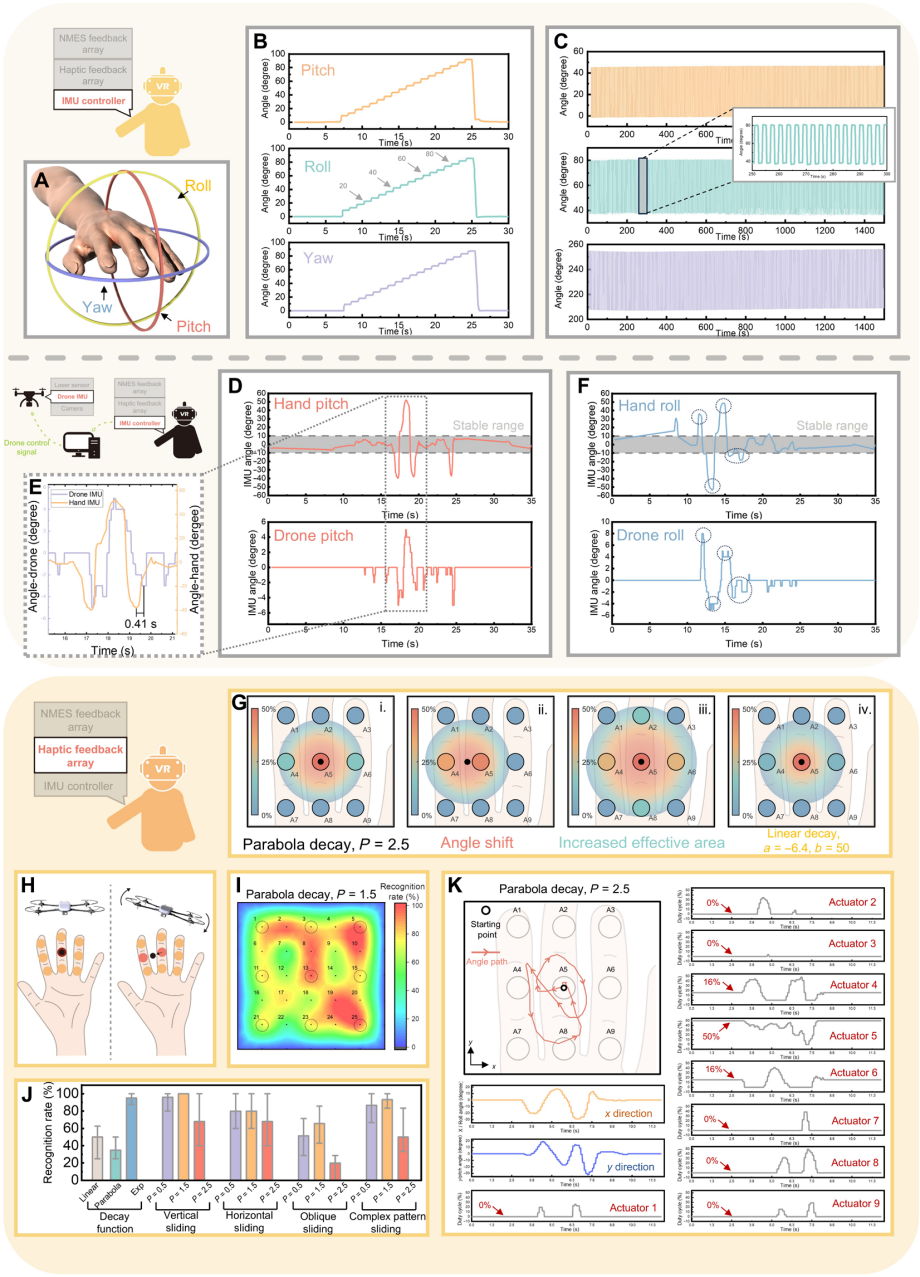
Performance of IMU on DCTF modules and evaluation of tactile feedback system. (**A**) Three rotation axes of the operator’s hand. (**B**) Assessment of the accuracy of the DCTF’s IMU across all three axes. (**C**) Evaluation of the long-term stability of the DCTF’s IMU. (**D**) Comparison of IMU on pitch axes during actual flight. Control command will only be activated when the hand rotation exceeds the stable range. (**E**) Detailed comparison of the IMU data. (**F**) Comparison of IMU on roll axes during actual flight; identical data shape can be observed. (**G**) Central point, effective area, and duty cycles of each actuator with original parabola decay function, angular variation, enlarged effective area, and linear decay function (from left to right). (**H**) Working principle of tactile feedback system. (**I**) Average recognition rate of single-point test of the tactile array integrated with downsampling algorithm and parabola decay with 1.5 focus. (**J**) Recognition rate of the user study with different decay function, sliding direction, and complex sliding pattern. (**K**) Dynamic duty cycle of each actuator during angular variation of the drone.

Given the system’s reliance on the orientation of the user’s hand as an intuitive means of drone control, the performance of the IMU within the DCTF modules shows critical importance in ensuring control quality. The three axes of rotation of the user’s hand (pitch, roll, and yaw) are depicted in Fig. 3A, which correspond to those of the drone. Rotation of the user’s hand will induce similar movements in the drone. For instance, after the calibration process that records the initial orientation of the user’s hand, if the user rotates their hand downward along the pitch axis, then the drone will likewise rotate in the pitch axis, causing the drone to fly forward. In the event that rotations on multiple axes of the user’s hand are detected (e.g., rotations on both the pitch and yaw axes), the drone will respond by flying with multiple vectors (e.g., forward and left). Moreover, a larger rotation detected by the DCTF modules prompts the drone to fly in the corresponding direction at a higher speed. Thus, the DCTF modules must be capable of accurately and reliably capturing angular variations in the user’s hand. Figure 3B displays the accuracy test results of the DCTF modules. The test is similar to that conducted on the drone, with results showing a notable 5° increment and a total 90° rotation observed for all three rotation axes. The 25-min long-term test results for the DCTF modules (Fig. 3C), exhibiting a consistent 45° variation, underscore the high repeatability of the DCTF system on angle monitoring. Similar to the drone’s IMU system, the DCTF modules also demonstrate high accuracy and repeatability, rendering them suitable for precise drone control. The angle variations of both the user’s hand and the drone in the pitch and roll axes during a real flight are illustrated in Fig. 3 (D and F). A detailed comparison of the pitch axis is presented in Fig. 3E. The similar patterns observed in both the user’s hand and the drone on these axes indicate that the system effectively converts the user’s commands regarding hand angle variations into flight commands for the drone, thereby guiding its movement. Although variations in the user’s wrist along the yaw axis can be detected and converted into angular rotations of the drone’s yaw axis, the relatively limited rotational range of the wrist in this direction necessitates mapping small wrist movements to large angular changes in the drone’s yaw axis. To enhance control stability, we therefore excluded the yaw axis from the final system design.

Tactile feedback has been integrated into the system, and its operational principle is elucidated in Fig. 3H. As established in the previous section, the drone’s angle during flight correlates with the surrounding aerodynamic conditions. This angle variation is replicated on the 3-by-3 tactile actuator arrays affixed to the user’s finger. When the drone tilts in a particular direction, the tactile feedback point also shifts from the center of the array toward the corresponding direction. This generates dynamic real-time tactile perception akin to a “rolling ball” on the user’s hand, enabling the user to perceive the aerodynamic conditions surrounding the drone. The configuration of an individual tactile actuator (10 mm diameter) used in the system is illustrated in fig. S11A. The cantilever structure on the polyethylene terephthalate film facilitates the unrestricted movement of the magnet, while a square wave signal with varying duty cycles (fig. S11B) is applied to the copper coil, inducing periodic upward and downward movement of the magnet and generating vibrations. The vibrational frequency of the actuators is adjustable, allowing the control circuit to output actuation signals at varying frequencies (fig. S12) to achieve different vibration performances. The resonant frequency of the actuator is examined (fig. S13A and movie S1), with vibration amplitudes compared across frequencies ranging from 45 to 185 Hz at intervals of 35 Hz. The results indicate that the actuator attains maximum vibration amplitude at 115 Hz, which is adopted in our system and considered the resonant frequency of the actuator. In addition, the impact of the duty cycle on vibration amplitude is investigated. Duty cycles ranging from 20 to 50% are applied to the actuator, revealing a strong proportional relationship between duty cycle and vibrational amplitude (fig. S13B and movie S2). This suggests that higher duty cycles result in greater vibration intensity. However, the maximum duty cycle adopted in our system is limited to 50%. This is because the longer duty cycle (>50%) will not yield a stronger vibrational amplitude compared to the 50% duty cycle ones but will consume more power. Infrared camera measurements are presented in fig. S14, illustrating the temperature dissipation of the actuator when operated at 5 V, 115 Hz, and a 50% duty cycle. Although the peak temperature of the coil in the actuator can reach 47.08°C (fig. S14A), the lateral view (fig. S14B) indicates that the temperature at the contact surface remains around 38.8°C, which is only slightly above body temperature. Thus, we conclude that the heating effect of the actuator is unlikely to cause any adverse effects on the user’s skin.

In order to replicate the angular changes of the drone to the user through tactile feedback, the alterations in the drone’s roll and pitch axis angles are extracted and plotted onto a virtual 2D map, generating a point designated as the “central point” (fig. S15A). Variations in the drone’s roll and pitch axis angles correspondingly change the *x* and *y* coordinates of the central point on the map. Because the angular changes of the drone should be continuous and analog, monitoring multidimensional angular changes in our system may lead to a multitude of potential combinations for tactile stimulation locations, posing a challenge if we allocate one exact actuator to each coordinate. For instance, monitoring angle changes from positive to negative 10° on both roll and pitch axes with 1° accuracy could yield 22-by-22 possible coordinate combinations, requiring replication by the tactile actuator array. This poses high demands on the density and resolution of the actuator array if each actuator represents only one coordinate on the map.

To regenerate informative spatial information on actuator arrays with lower resolution, we introduce and evaluate a spatial downsampling method in our 3-by-3 tactile feedback arrays. In our design, the distance (∆*d*) between each actuator and the central point is calculated and inputted into a decay function, determining the duty cycle of each actuator based on ∆*d*. If the result is positive, then the corresponding actuator will be activated with the calculated duty cycle, while a negative result renders the actuator idle. Using this method, a circular area is delineated on the virtual feedback map surrounding the central point, where actuators are activated upon entering this area and stimulation intensity gradually increases until reaching the maximum strength at the central point. This area is referred to as the “effective area” (fig. S15B). As the drone undergoes angular variation, the central point, along with the effective area, moves correspondingly (Fig. 3K and fig. S15C), altering the duty cycle of each actuator. Consequently, continuous angular variation is downsampled and reproduced through changes in the stimulation intensity of each actuator.

There are multiple parameters in the downsampling method that can affect the tactile feedback experience and the efficiency of information transmission, as depicted in Fig. 3G (graphical representation) and fig. S16 (decay function representation). For instance, starting with the original parabolic effective area [Fig. 3G(i) and fig. S16A], the position of the effective area can shift according to the angular variations of the drone [Fig. 3G(ii) and fig. S16B], and the size of the effective area can be expanded or reduced by adjusting the parameters in the decay function [Fig. 3G(iii) and fig. S16C]. In addition, the decay function can be altered to generate different types of dynamic tactile feedback, such as transitioning from a parabolic function to a linear decay function [Fig. 3G(iv) and fig. 16D]. To assess the impact of different parameters, we conducted user studies. Five volunteers were invited to wear the DCTF module for testing, and the results are presented in Fig. 3J. First, responses to various decay functions were explored, comparing three decay functions: parabolic, linear, and natural exponential function (fig. S17A). Different decay functions elicited diverse dynamic responses in the duty cycle, as the central point approached the actuator on the virtual map. Volunteers were presented with three levels of feedback intensity (low, moderate, and high stimulation) and asked to identify the intensity level. Results revealed that the natural exponential decay achieved the highest recognition rate of 95%, surpassing the linear decay, while the parabolic decay exhibited the lowest recognition rate. Among the selected feedback intensity range, the natural exponential decay curve exhibited the highest cumulative slope, indicating the most marked variation in stimulation intensity, which may account for its high recognition rate. Then, nine actuators on different fingers were activated at the highest intensity (50% duty cycle), and volunteers were asked to locate the vibrating actuator. The high recognition rate (fig. S18) suggests that, even at the highest stimulation intensity, mechanical cross-talk among different actuators is minimal and unlikely to affect the perception of haptic feedback. Subsequently, comparisons were made between different effective areas in various tactile feedback patterns. During testing, three different effective areas, ranging from small to large, were evaluated (fig. S17B). In the “single-point test”, the central point was randomly positioned at 25 locations within the virtual map (fig. S19D), and volunteers were tasked with identifying the central point’s location. The effective area generated by the 1.5 focus (*P* = 1.5) demonstrated the most promising outcome with the highest accuracy rate (Fig. 3I). Conversely, the 0.5 focus (*P* = 0.5) (fig. S20A), while achieving high recognition rates at actuator locations, exhibited a noticeable drop in areas between actuators, resulting in four “blind spots” (locations 7, 9, 17, and 19) due to the excessively small effective area that failed to trigger any actuators in those locations. In addition, the 2.5 focus (*P* = 2.5) (fig. S20B) yielded the poorest performance, possibly due to the larger effective area, causing confusion and difficulty for volunteers in locating the central point. Results from the “sliding test,” which perform sliding of the central point in different basic sliding patterns (fig. S19, A to C), aligned with those of single-point test, with the 1.5 focus also achieving the highest recognition rate among all three effective areas. In recognizing complex patterns (fig. S19E), which are most akin to the actual application scenario of the drone’s closed-loop feedback, the 1.5 focus yielded a high recognition rate of 93.3%. Therefore, the natural exponential decay function and effective area generated by a focus of 1.5 in the parabolic function were adopted because of their outstanding performance in the user study, and the user study confirms that the 3-by-3 tactile feedback arrays, along with the spatial downsampling method, are capable of effectively transmitting 2D spatial information to users for monitoring the drone’s flight posture.

**Fig. 4.**
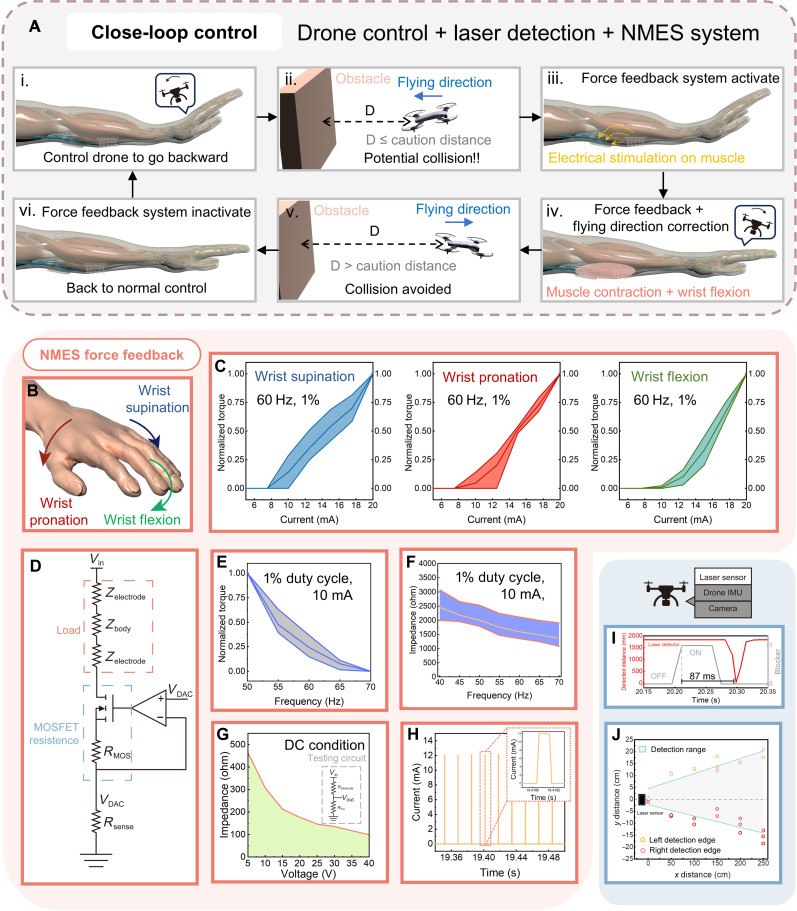
NMESF system and obstacle detection system. (**A**) Application scenario of the NMESF system collision avoidance. (**B**) Three rotational directions of the operator’s hand induced by NMES. (**C**) Relationship between stimulation current and torque in all three bending directions. (**D**) Equivalent circuit of the current stimulation pathway. (**E**) Relationship between stimulation frequency and generated torque on wrist flexion. (**F**) Relationship between stimulation frequency and measured impedance of the stimulation path on wrist flexion. (**G**) Impedance response of stimulation electrode under different voltage. (**H**) Stimulation current pulse used in NMES. (**I**) Response of the obstacle detection system when an obstacle is detected. (**J**) Detection angle and range of single laser detector.

The force feedback system constitutes a crucial component of the overall system. While visual feedback has been established as an effective means for the drone to aid in stable flight, it is limited in providing all-angle coverage. Even if the drone were capable of capturing visual signals from all angles, the constrained visual perspective of the human operator would still present challenges. This limitation becomes even more pronounced for drones compared to conventional fixed-wing aircraft, as drones have the ability to fly in various directions, including left, right, and backward. To address this issue, we incorporated an obstacle detection and force feedback system into our design. Figure 4A shows a typical application scenario of the system. During control, if the user raises their wrist, causing the drone to move backward due to the intuitive gesture control, then the absence of rearward visual information from the drone may prevent the user from detecting obstacles positioned behind the drone. Consequently, continued backward movement of the drone could lead to a collision. To mitigate the risk of collision, we transmitted a stimulation command to the NMESF modules upon detection of an obstacle by the obstacle detection system within the system’s caution distance. Leveraging the principle of NMES, electrical stimulation is delivered to the user’s muscles (flexor carpi ulnaris and flexor carpi radialis) and induces muscle contraction. As the drone approaches the obstacle, the intensity of stimulation gradually increases, causing stronger muscle contraction and wrist flexion in opposition to the upward motion of the wrist. This involuntary wrist flexion results in forward movement of the drone, counteracting the impending collision. The force feedback system is inactive and stimulation ceases when the drone moves beyond the caution distance. Thus, the force feedback system not only enables users to perceive invisible obstacles but also assists in course correction to avert potential collisions. Unlike traditional force feedback devices reliant on exoskeletons or bulky devices, the force in our system is generated from the user’s muscles. Consequently, only thin electrodes and peripheral circuits are necessary, facilitating the miniaturization of the entire device to a size that allows the wearing of the device under everyday attire (fig. S9). The extensive historical background and broad application of NMES in rehabilitation and neuroprosthetics (*54*–*56*), as well as in muscle strengthening (*57*–*60*) and human-machine interaction (*61*–*63*), demonstrate the safety and reliability of the NMES technique for use on the human body (*64*, *65*).

Three groups of muscles (flexor carpi ulnaris and flexor carpi radialis for flexion, pronator teres for pronation, and supinator for supination) (*66*) will be stimulated by five electrodes, as depicted in fig. S21, inducing contractions that produce rotation of the wrist in three directions: pronation, flexion, and supination (Fig. 4B). These rotations occur along the roll and pitch axes of the user’s hand, which are used in intuitive drone control (Fig. 3A), and are capable of counteracting the left, right, and upward rotations of the user’s wrist. Electrical stimulation will be generated by the NMESF modules in the form of electrical current pulses (Fig. 4H), and the NMESF system is capable of delivering pulses with varying current levels (fig. S22A), duty cycles (fig. S22B), and frequencies (fig. S22D) through the current control circuit by adjusting the digital-to-analog (DAC) output of the MCU. The current regulation circuit is depicted in Fig. 4D. The DAC output from the MCU is input into an operational amplifier (OP-AMP), which automatically adjusts the resistance of the MOSFET [*R*_ds(on)_]. As a result, the voltage at the inverting input matches the voltage output from the DAC. According to Ohm’s law, the current flowing through the sensing resistor can be determined. Considering that this current also flows through the user’s muscle, the stimulation current can be effectively regulated. A straightforward control is established because of the linear relationship between the DAC output and the stimulation current output (fig. S22C). To assess the force generation capability of NMES on the user’s muscles, torque tests were conducted on the testing platform (fig. S23), with the results displayed in Fig. 4C. From the results, it is evident that although different users may exhibit different stimulation thresholds, the average torque generated during both supination, pronation, and flexion exhibits a proportional or even linear relationship with the stimulation current, demonstrating that the torque and force applied to the user’s wrist by their own muscles can be accurately manipulated by adjusting the stimulation intensity. The motivation thresholds from different volunteers are presented in fig. S24. While individual differences and variations among different muscle groups are observed, the motivation threshold generally remains around 9 mA. The unnormalized torque-current data of the three volunteers are presented in fig. S25, where it is noteworthy that even the weakest wrist pronation can produce a torque around 0.4 Nm when high current is applied to the user’s muscle, and this notable torque can induce involuntary wrist bending in the corresponding direction, aiding in path correction during flight.

Figure 4D illustrates the equivalent circuit of the stimulation pathway, where the load refers to the impedance of the user’s muscle and the impedance of the electrodes. According to the equivalent circuit, it is evident that if the load impedance is high, then a higher input voltage generated by the voltage boost circuit (*V*_in_) will be required on the basis of Ohm’s law. Therefore, the electrical characteristics of the electrodes and the user’s body become critical factors in reducing the requirement on the voltage boost circuit. One possible solution is to increase the stimulation frequency. Figure 4F displays the average frequency response of the load, which includes two electrodes and the user’s muscle, under electrical stimulation. A noticeable decrease in average impedance from 2450 to 1360 ohms is observed as the stimulation frequency increases from 40 to 70 Hz. This may be attributed to the same decreasing trend in the frequency response of the electrode as the frequency increases (fig. S26), and human body impedance may also exhibit a declining response to the increasing frequency (*67*–*69*). However, testing indicates that the torque generated by the user also decreases with the increasing frequency (Fig. 4E). To balance the impedance of the stimulation path and the generated torque, we selected 60 Hz as our final stimulation frequency. Because the hydrogel is designed to be replaceable, it is removed from the electrode surface after each use. The long-term stability of the electrode was also tested by leaving the hydrogel on the electrode surface for 1 week. According to the results (fig. S27), the electrical characteristics of the electrode remained stable, with only a 17.44-ohm decrease in resistance observed. Given that the impedance of the user’s muscle is on the order of thousands of ohms, this result suggests that the electrode exhibits excellent long-term stability for electrical stimulation applications. While the system demonstrates the capability to generate current pulses with diverse duty cycles, users have reported experiencing discomfort as the duty cycle increases. Consequently, the duty cycle is maintained at 1% throughout the stimulation to mitigate any discomfort for the user.

Despite optimizing the impedance of the stimulation pathway by adjusting the stimulation frequency, a high voltage remains necessary to overcome the 1.6-kilohm average impedance of the load at 60 Hz. In addition, the relationship between excitation voltage and the impedance of the electrode (Fig. 4G) indicates that a higher stimulation voltage can further decrease the impedance of the electrode. This finding led to the design of a two-stage voltage boost circuit in the NMESF module. The boost circuit is designed to elevate the input of 7.4 V from batteries to an output of 100 V for electric stimulation. The power conversion efficiency and output capacity of the boost circuit are illustrated in fig. S28. The selection of a 7.4-V input from the battery and a 15-V output from the first stage of the boost circuit aims to maximize power conversion efficiency, as shown in fig. S28 (B and C). The power conversion efficiency can reach approximately 75% when the boost circuit outputs 15 mA (fig. S28D). Despite limiting the battery input voltage to 6.8 V to mimic low-battery conditions, the maximum output power of the system can achieve 1.78 W. Beyond 1.78 W, while the output current may continue to increase, the output voltage drops, resulting in only a slight overall increase in power, which remains at the 1.8-W level (fig. S28G). We believe that the limiting factor is the first stage of the boost circuit, which exhibits a voltage drop after 1.8 W (fig. S28E). However, even with a voltage drop occurring when the boost circuit outputs 20 mA (the maximum output current set by our system), the remaining 89-V supply is sufficient to maintain stable current output for our force feedback system. Although the maximum power of the stimulation system can reach 1.8 W, the duty cycle of the stimulation pulse is only 1%, meaning the system does not output its maximum power. As a result, not all of the 1.8 W is delivered to the user’s body. Furthermore, as shown in the infrared camera measurements (fig. S29), no notable heating effect was observed on the circuit, and it is unlikely to cause any discomfort when mounted on the user’s skin. The battery is capable of sustaining operation for 265 min when the system outputs a 10-mA current with a 1000-ohm load. We believe that this duration is sufficient to support the system’s daily operation. Furthermore, we evaluated the performance of the obstacle detection system. Figure 4I and fig. S30A demonstrate that the system can detect obstacles with a short response time of 87 ms, which is feasible for scenarios involving drones or obstacles moving at high speeds and requiring rapid reactions. Figure S30B illustrates the accuracy of the system in detecting obstacles at different distances, while Fig. 4J demonstrates the system’s ability to detect obstacles up to 2.5 m away. In addition, the median detection range enables the system to effectively detect obstacles without interference from obstacles in other directions.

**Fig. 5.**
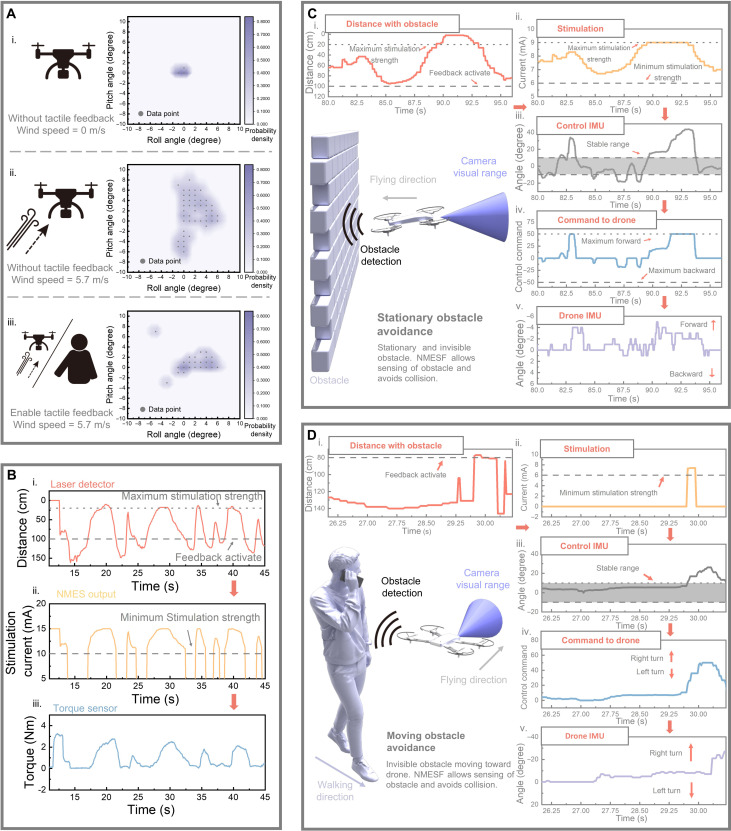
Demonstration of performance under actual flight. (**A**) Comparison of angular variation in windy conditions with and without the tactile feedback system. Abnormal angular variation is perceived by the user via tactile feedback, prompting corrective adjustments to the flying posture. (**B**) Relationship between obstacle detection system, stimulation intensity, and torque generated by the user under wrist flexion conditions. (**C**) User response data when a stationary rearward obstacle is detected during flight. (**D**) User response data when an obstacle moving from the side is detected during flight.

In Fig. 5, we present the performance of our multimodal closed-loop human-drone interface system in an actual flying scenario. Figure 5A illustrates how the tactile feedback system enhances the control stability of the drone. It displays the angular variation of the drone under windy conditions with and without the tactile feedback system. The *x* and *y* axes represent the roll and pitch rotation of the drone, respectively, while the depth of color indicates the density of data points. Under windless conditions, the drone remains stable with a variation of 1° to 2° [Fig. 5A(i)]. However, when subjected to a wind speed of 5.7 m/s, the angular variation rapidly increases [Fig. 5A(ii)]. Upon activating the tactile feedback system, users are able to perceive the abnormal changes in the drone’s posture and counteract the wind, resulting in lower angle changes and a more stable flight [Fig. 5A(iii)]. This demonstrates that the tactile feedback system can enhance the flying stability of the drone under complex aerodynamic conditions. The ability of the obstacle detection system to trigger muscle contraction through NMESF modules is validated in Fig. 5B. The system is configured to activate stimulation when an obstacle is detected within 1 m. Stimulation strength gradually increases as the obstacle approaches, reaching a maximum strength of 20 cm. The data indicate a consistent correlation between obstacle distance [Fig. 5B(i)], stimulation current [Fig. 5B(ii)], and torque generated by the muscle [Fig. 5B(iii)], confirming the force feedback system’s capability to contract user muscles upon obstacle detection. Figure 5 (C and D) demonstrates the drone’s invisible obstacle avoidance ability when the force feedback system is activated. Following the initial calibration process (fig. S30), users control the drone’s flight and encounter obstacles. Figure 5C and movie S3 depict the system’s ability to avoid stationary obstacles behind the drone, which are invisible in the drone’s FPV. The system detects and generates stimulation, providing force feedback to counteract user wrist movements and correct the flight path, as evidenced by IMU data from both the user’s hand and the drone, which avoid the upcoming collision. Similarly, Fig. 5D and movie S4 show the system assisting users in quickly avoiding invisible obstacles moving sideways relative to the drone. An obstacle avoidance action is observed in the user’s hand motion after the stimulation generates force feedback without seeing the obstacle, resulting in a change in the drone’s flight path and the avoidance of potential collisions from unexpected angles. The response times for the NMESF and DCTF feedback pathways are 110 and 90 ms, respectively. Notably, as shown in Fig. 5D, the system requires only 0.6 s from obstacle detection to the drone initiating a change in its flight path. We believe that the short system response time, coupled with the rapid user reaction time demonstrated during actual flight tests, highlights the potential of our system for applications involving high-speed drone operations or fast-moving obstacles.

## DISCUSSION

In conclusion, we have developed a thin, soft, stretchable, and skin-conformal multimodal closed-loop system for human-drone interaction. This system incorporates NMES-based force feedback, 2D tactile feedback with a downsampling method, FPV visual feedback, and intuitive gesture control of the drone, forming multiple control feedback loops. Through comprehensive studies on the mechanical performance of device, accuracy and reliability of user hand and drone orientation monitoring, mechanical performance of vibrational actuators, recognition rate of tactile feedback array, and downsampling method, as well as mechanical and electrical performance of the force feedback system, we have demonstrated improvements in flight stability and the ability to avoid invisible obstacles. By applying this system in drone control, we have enhanced the drone’s capability to navigate complex and dynamic environments.

## MATERIALS AND METHODS

### Stretchable circuit and actuator fabrication

The fabrication process of the stretchable circuit begins with the preparation of a piece of quartz glass (75 mm by 75 mm), which is thoroughly cleaned with acetone, alcohol, and deionized water to serve as the supporting layer. A sodium stearate aqueous solution is then spin coated onto the glass and dried at 100°C for 5 min, forming a thin sacrificial layer that facilitates the later removal of materials. Subsequently, 3.5 ml of a 20:1 mixture of PDMS and white silicone (Silc Pig) at a weight ratio of 1:310 is spin coated onto the glass substrate at 500 rpm for 30 s. The coated substrate is then baked at 110°C for 6 min. Spin coating and baking are repeated again with the same parameter to form a 400-μm thin film of PDMS, which serves as the stretchable substrate for the device. Following this, a copper and polyimide (PI) film is affixed onto the PDMS film. Another baking step at 110°C for 6 min is carried out to ensure strong adhesion between the copper and PI circuit layer and the PDMS substrate. Subsequently, the plate undergoes laser cutting using the ProtoLaser U4 from LPKF (fig. S4). Initially, the laser cutter shapes the circuit, followed by the removal of excess copper and PI layers. After that, the plate is spin coated with 3.5 ml of a 20:1 PDMS substrate at 700 rpm for 30 s and baked at 110°C for 12 min to create the encapsulation layer. The plate will undergo laser cutting once more to outline the device and create soldering pads on the surface. For double-layer circuits, the vias are also cut using a laser cutter. The two layers are aligned and assembled with an ultrathin PDMS layer in between to create a strong adhesive bond between the layers. Vias are interconnected and soldered using copper pins with a diameter of 0.4 mm. MOSFET (BSS138BKVL) is soldered on the soldering pad using a low-temperature soldering paste. For the fabrication of the electrode used in electrical stimulation, the PI + Cu conductive layer is initially patterned using a laser cutter. After removing the undesired regions, a water-soluble tape is used to transfer the conductive layer onto the PDMS substrate of the electrode. To achieve a robust adhesion between the PI surface of the conductive layer and the PDMS substrate, silicone adhesive (988A, JUKAM) is applied to the PI surface before the transfer. This step is crucial for preventing delamination of the conductive layer from the substrate during deformation. Last, the assembled electrode is immersed in water to dissolve the water-soluble tape, thereby completing the transfer process.

### Electrical hardware

The DCTF control unit (fig. S32A) and the NMESF control unit (fig. S32B) are based on FPCB, MCU (STM32F410CBT3), Bluetooth module (WH-BLE106), low-dropout regulator (AP7370), resistor (0603), and capacitor (0603) adopted by both the DCTF control unit and the NMESF control unit. The DCTF control unit integrated IMU (BNO080). The NMESF control unit includes an analog switch (HV2621/RX4), OP-AMP (LT1784IS5#TRPBF), MOSFET (FDT86246L), and boost circuit controllers (XC9144B10CER-G and LT8331EMSE#PBF). All components are soldered on the FPCB using a low-temperature soldering paste. The obstacle detection system (fig. S32C) mounted on the drone (Tello, DJI) is based on PCB, including MCU (STM32F405RGT6), low-dropout regulators (AP7370 and TLV76650QWDRBRQ1), three laser detectors (TF-Luna, DFRobot), RF communication module (UltiRobot), resistor (0603), and capacitor (0603). All components are also soldered using a low-temperature soldering paste.

### Communication protocol

The communication protocol is shown in fig. S7. IMU data from the DCTF module are sent to the base station via Bluetooth mesh with 13-byte information including the roll, pitch, and yaw angle. Base station translates the IMU data into a 3D control command and delivers them to the drone using WLAN. The roll, pitch, and yaw angle data of the drone are sent back to the base station, are translated into actuator command based on decay function, are packed into 16-byte data, contained the duty ratio of each actuator, and are sent back to the DCTF module using Bluetooth mesh. The obstacle detection system sends a 5-byte message to the base station that contains the distance between the obstacle and the identity number of the sensor using RF. Base station translates the message into electrical stimulation command and delivers the command to NMESF modules with a 12-byte message containing the current, frequency, and targeted channel by Bluetooth mesh.

### Data collection

IMUs of the DCTF module and the drone are tested using a custom robotic arm, rotated from 0° to 90° with 5° increment for accuracy test and periodic 45° for long-term stability test. The windy environment for angular variation test on the drone under windy conditions is produced by a fan, in which wind speed is tested by a wind speed sensor (HT9829, Xintai Instrument Co.). For the user study on actuator array and NMESF, five volunteers are invited. The volunteers will wear the DCTF modules first for the decay function test, single-point test, sliding test (vertical sliding, horizontal sliding, and oblique sliding), and complex pattern recognition test. After that, the volunteers take off the DCTF modules, put on the NMESF modules, and undergo the calibration program (fig. S31). Torque test is conducted on a custom torque testing platform (ZNNF-20 Nm, CHINO sensor) that can be reassembled in two formats to supination, pronation, or flexion. The electrical performance of the electrode and human is tested with testing circuit attached on data graph and recorded with an oscilloscope (DS1202, RIGOL). Output ability of the boost circuit is tested by an electronic load (IT8512H+, ITECH). The laser detector is tested using a Stepper motor (Tr8, FUYU).
