# Supplementary material for: Skin-interfaced multimodal sensing and tactile feedback system as enhanced human-machine interface for closed-loop drone control
Source: Sci Adv. 2025 Mar 26;11(13):eadt6041. doi: 10.1126/sciadv.adt6041 (PMC11939050; doi:10.1126/sciadv.adt6041)
Supplement: Supplementary file 1 — Figs. S1 to S32 Legends for movies S1 to S4 [file sciadv.adt6041_sm.pdf]

Science Advances
AAAS

Supplementary Materials for

Skin-interfaced multimodal sensing and tactile feedback system as enhanced human-machine interface for closed-loop drone control

Chunki Yiu *et al.*

Corresponding author: Xinge Yu, xingeyu@cityu.edu.hk; Qingsong Xu, qsxu@um.edu.mo

Sci. Adv. **11**, eadt6041 (2025)
DOI: 10.1126/sciadv.adt6041

The PDF file includes:

Figs. S1 to S32
Legends for movies S1 to S4

Other Supplementary Material for this manuscript includes the following:

Movies S1 to S4

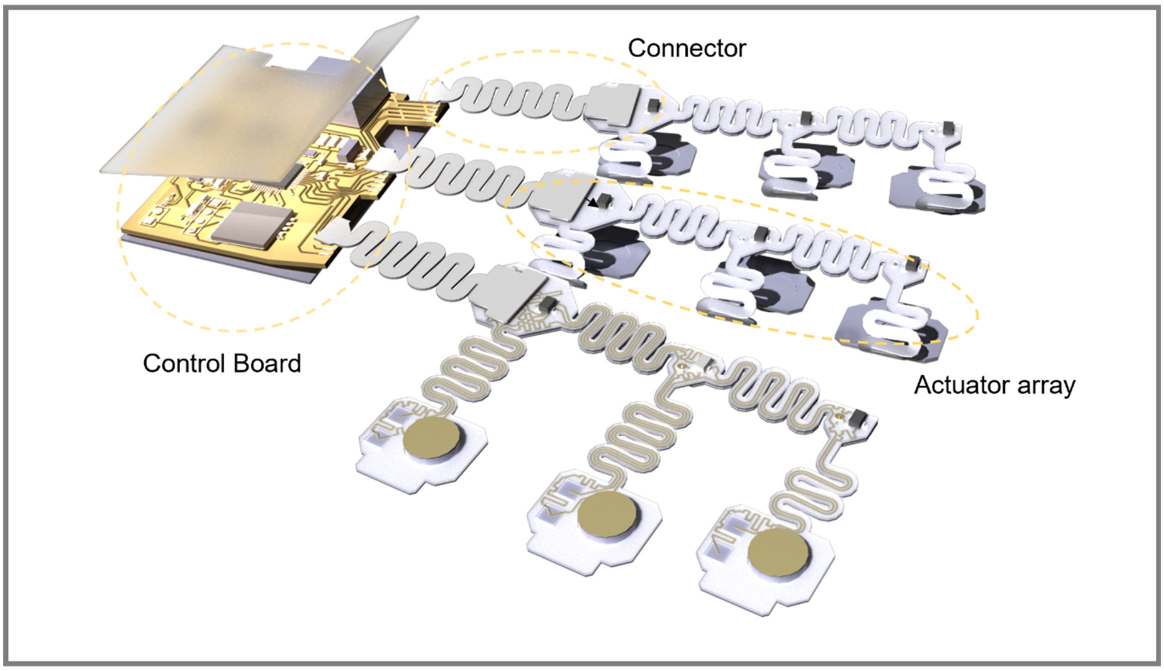

Fig. S1.
Structure of the DCTF modules.

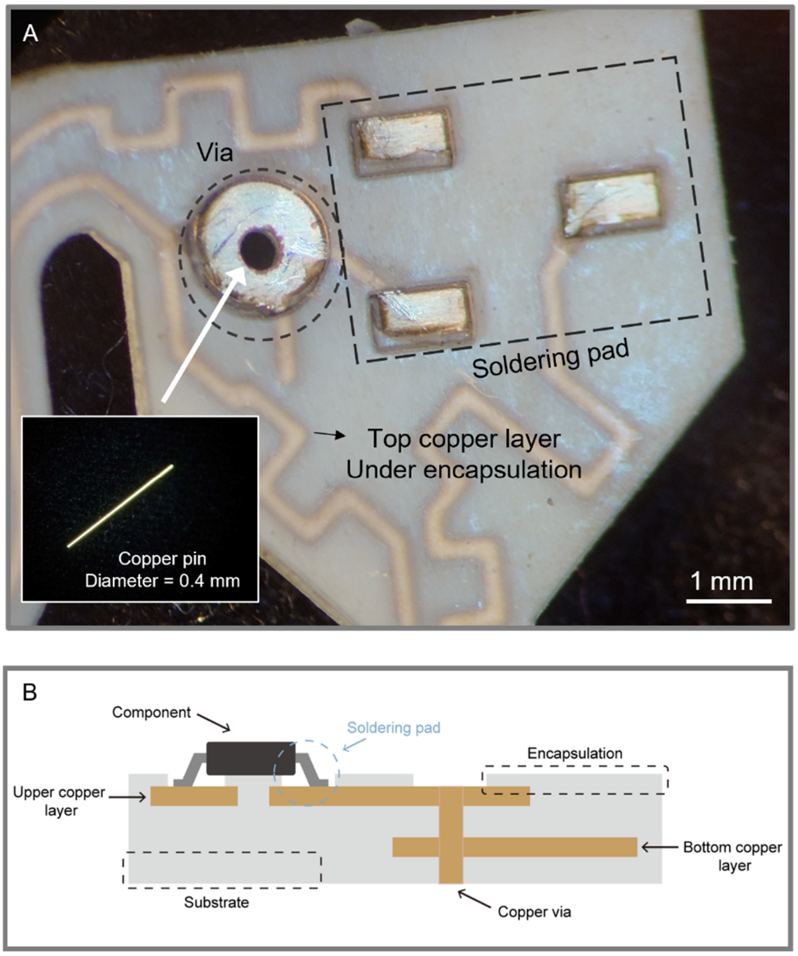

Fig. S2.

Structure of the double-layer stretchable circuit. (A) Optical image of the Encapsulation layer, soldering pad and via. **(B)** Lateral view of the double-layer structure.

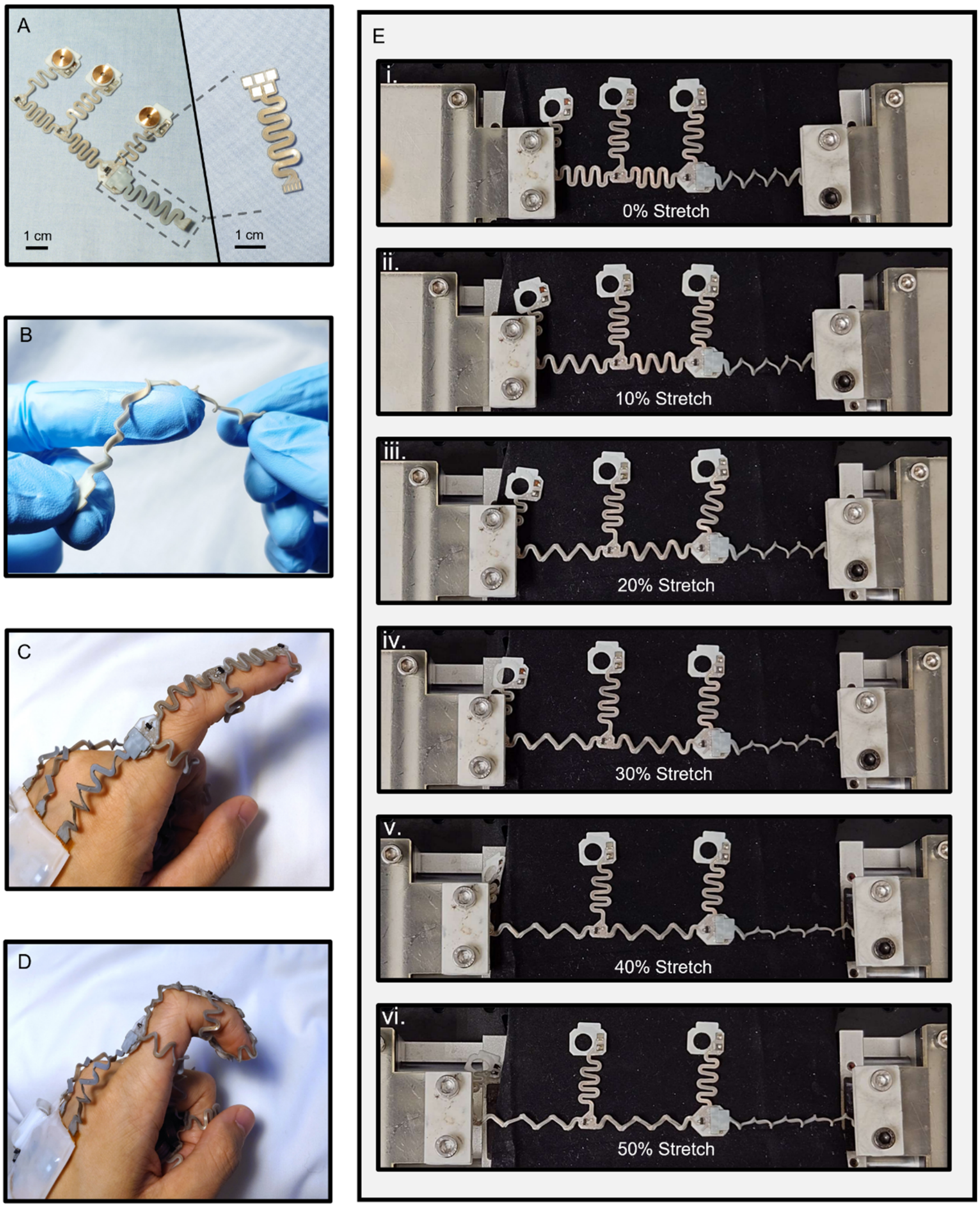

Fig. S3.

Stretchability of the actuator array. (A) Optical image of the tactile actuator array and connector. **(B)** Stretching of the serpentine structure of the connector. **(C)** Connector and tactile actuator array mounted on user's finger**. (D)** Bending of the finger with the stretchable circuit. **(E)** Stretching test conducted on the tactile actuator array.

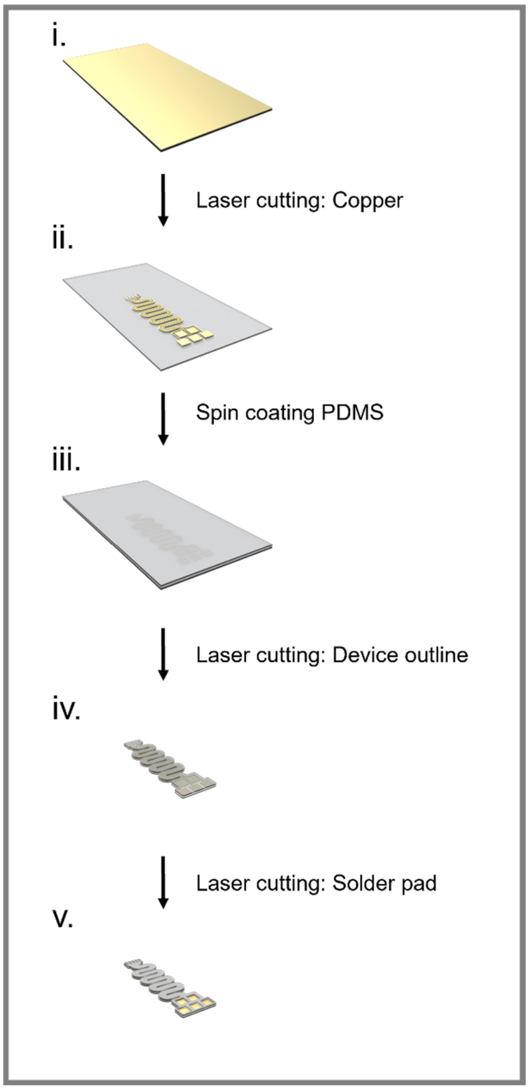

Fig. S4.
Fabrication procedure of the stretchable circuit.

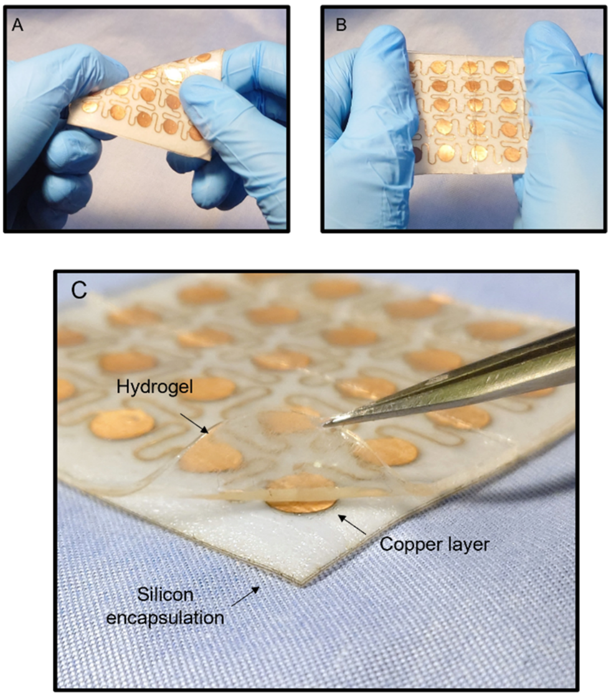

Fig. S5.

Optical view of the stimulation electrode. (A) Twisting of the electrode. **(B)** Stretching of the electrode. **(C)** Optical view of the three-layer electrode configuration.

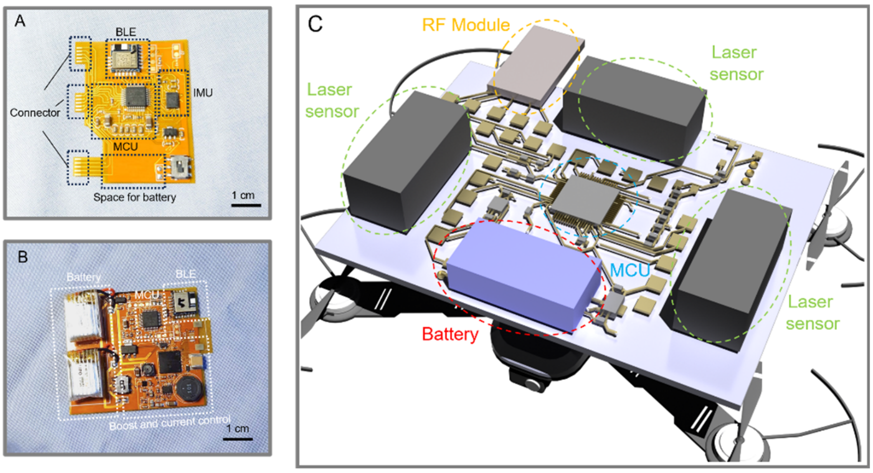

Fig. S6.

Electrical circuit board (A) Optical view of the DCTF control unit without encapsulation. **(B)** Optical view of the NMESF control unit without encapsulation. **(C)** Structure of the obstacle detection system circuit board.

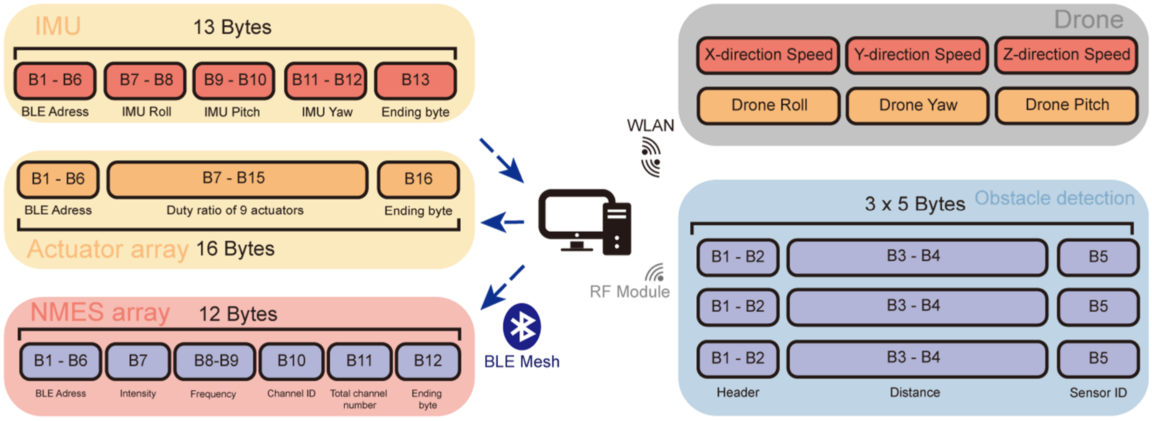

Fig. S7.

Communication protocol of the system.

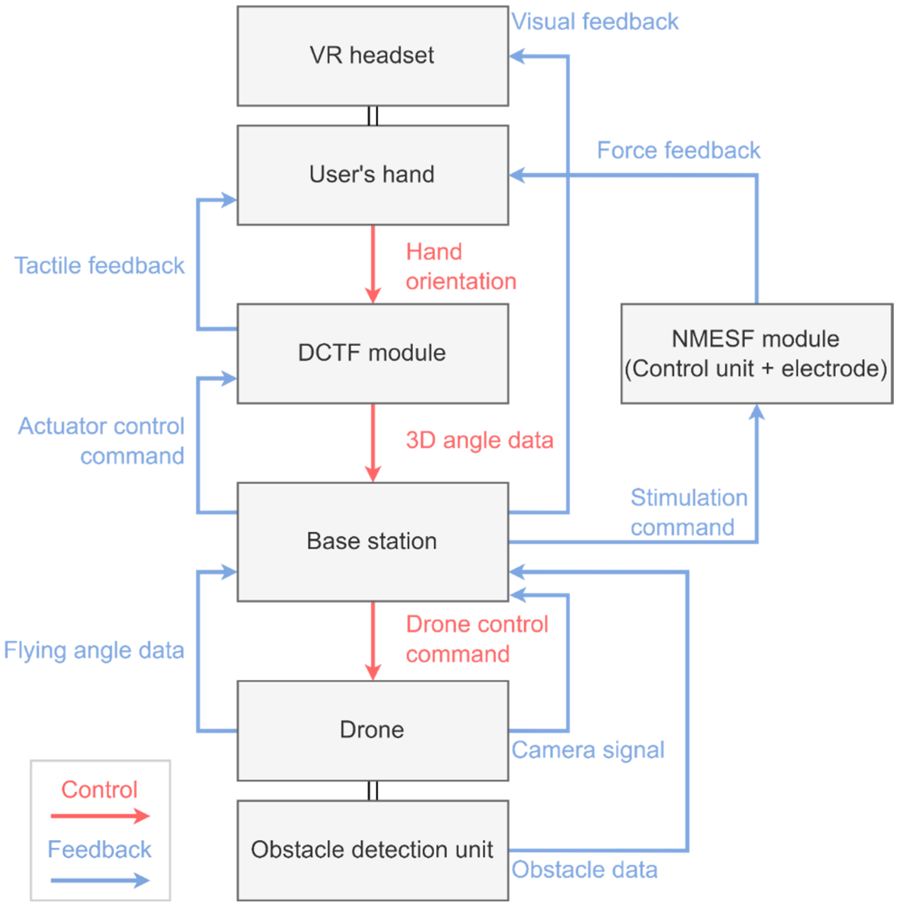

Fig. S8.

Logic diagram of the multimodal closed-loop system.

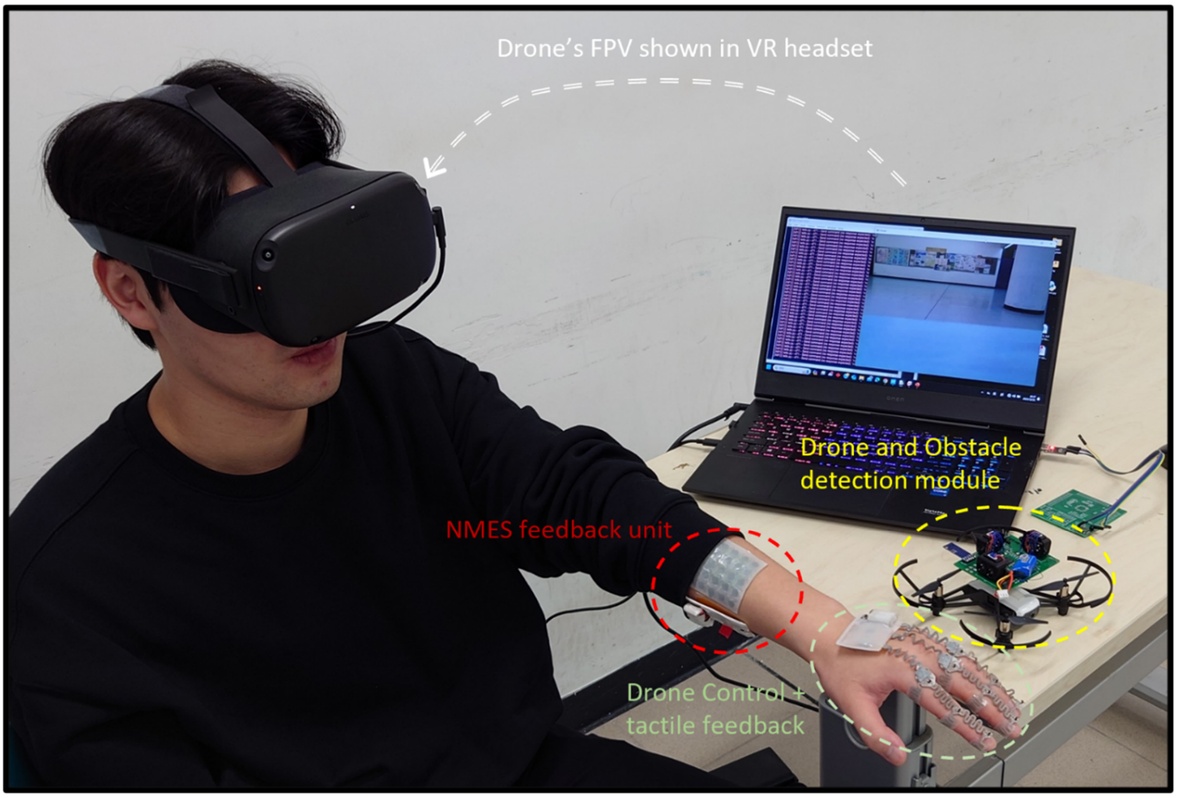

Fig. S9.
Optical image of user wearing the multimodal closed-loop system and VR headset.

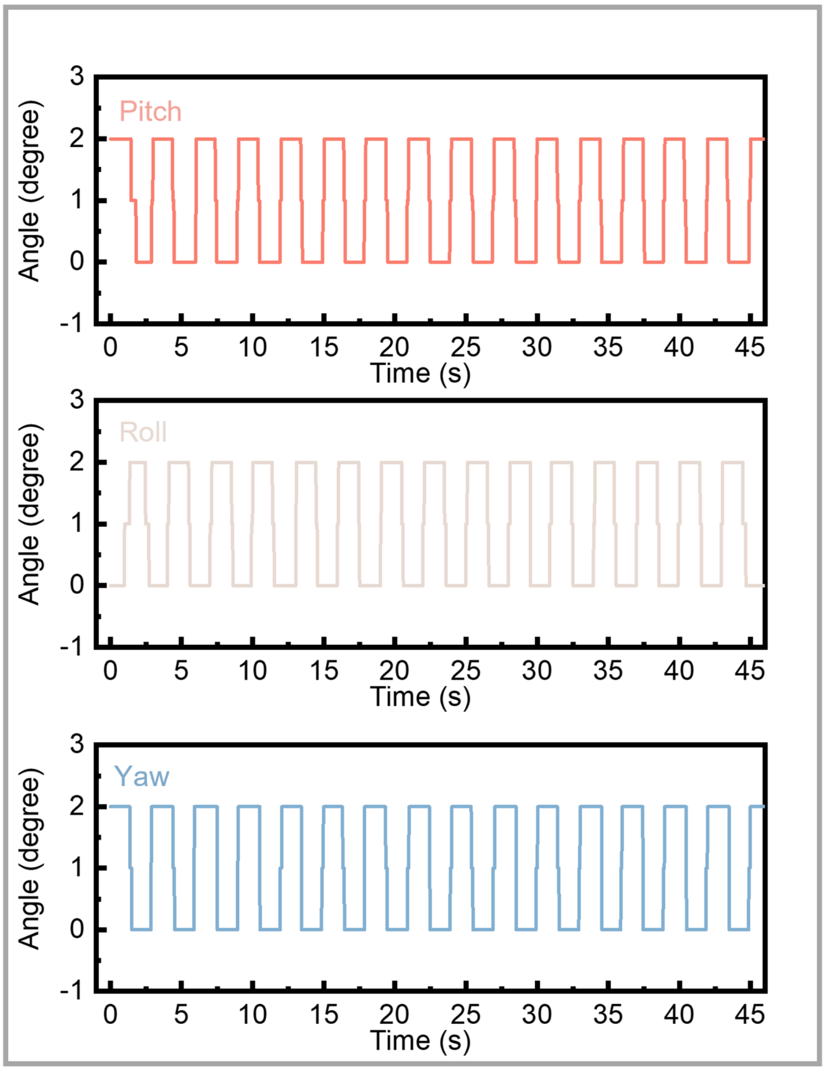

Fig. S10.

Repeatability assessment of the drone's IMU under small-degree variation conditions.

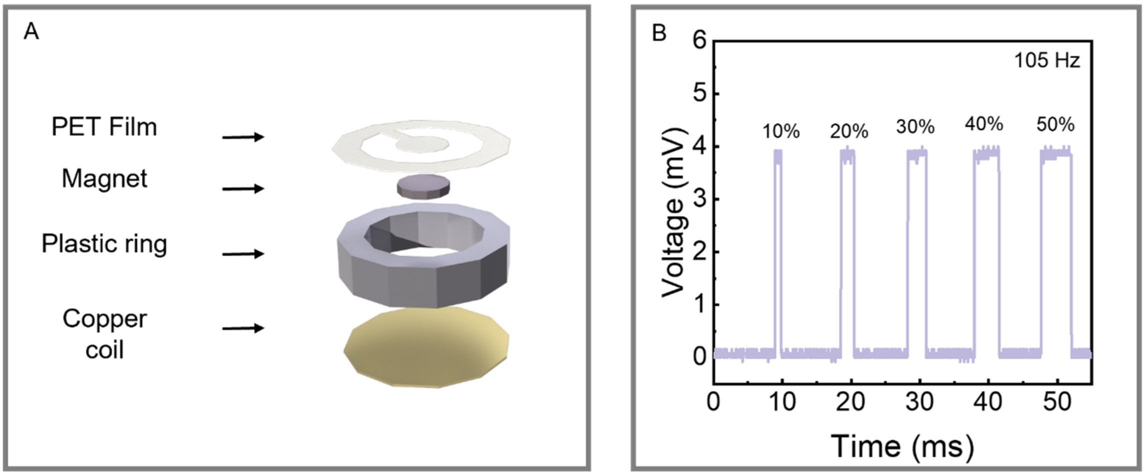

Fig. S11.

Tactile feedback actuator. (A) Structure of a single tactile actuator. **(B)** Pulse-Width Modulation signal used to control the vibration of actuator with different duty cycles.

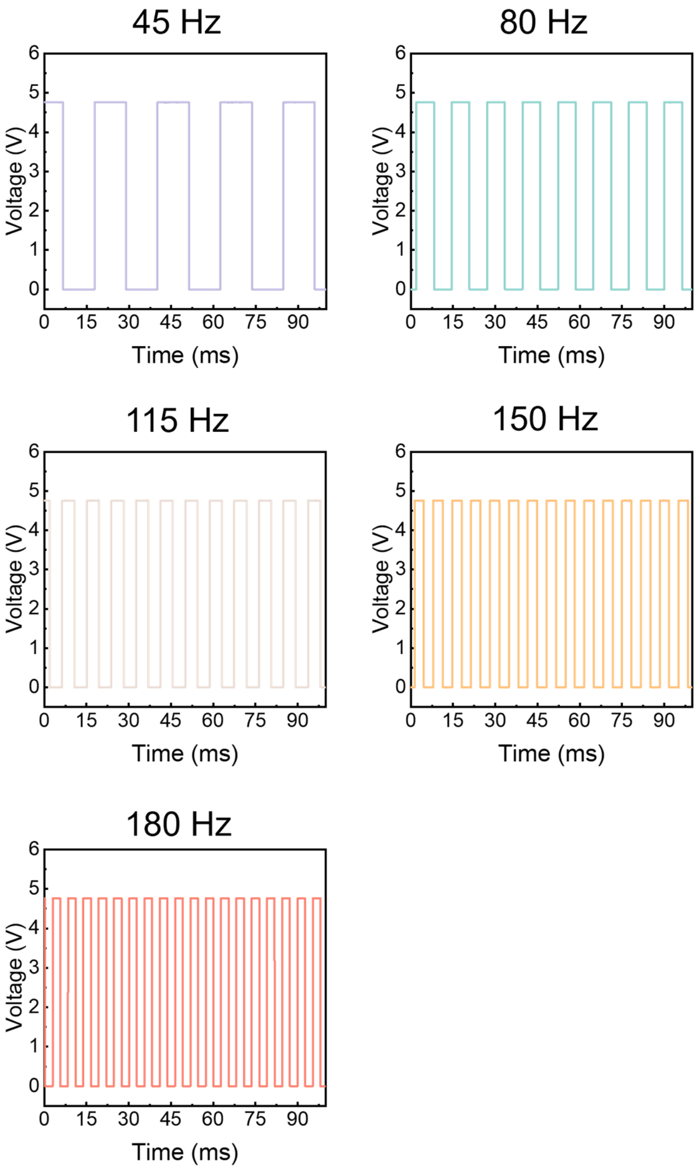

Fig. S12.

Actuation signal for vibrational actuators from DCTF control circuit with different frequencies.

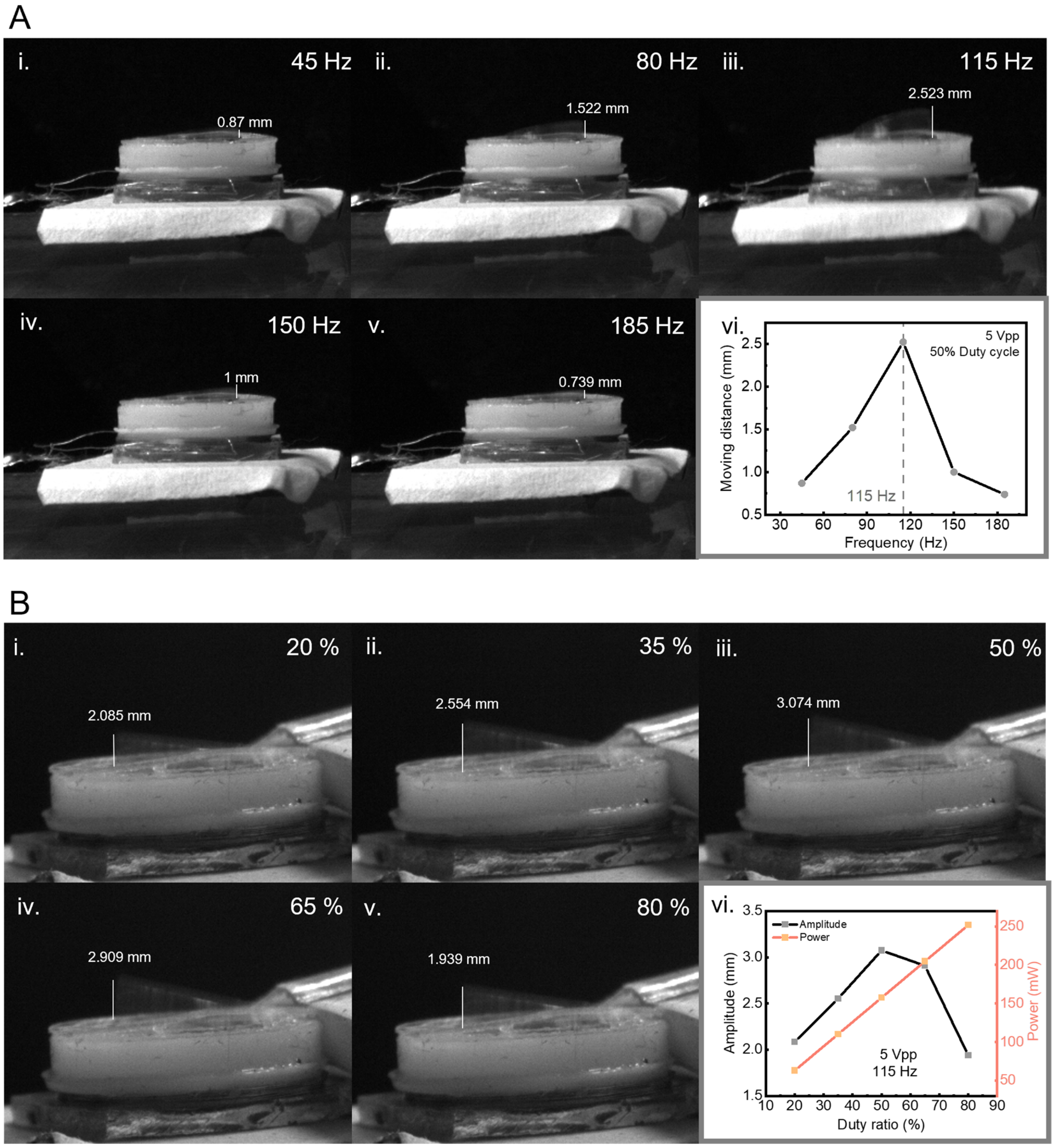

Fig. S13.

Mechanical performance of actuator. (A) Amplitude of vibration under different frequencies. **(B)** Amplitude of vibration under different duty cycles.

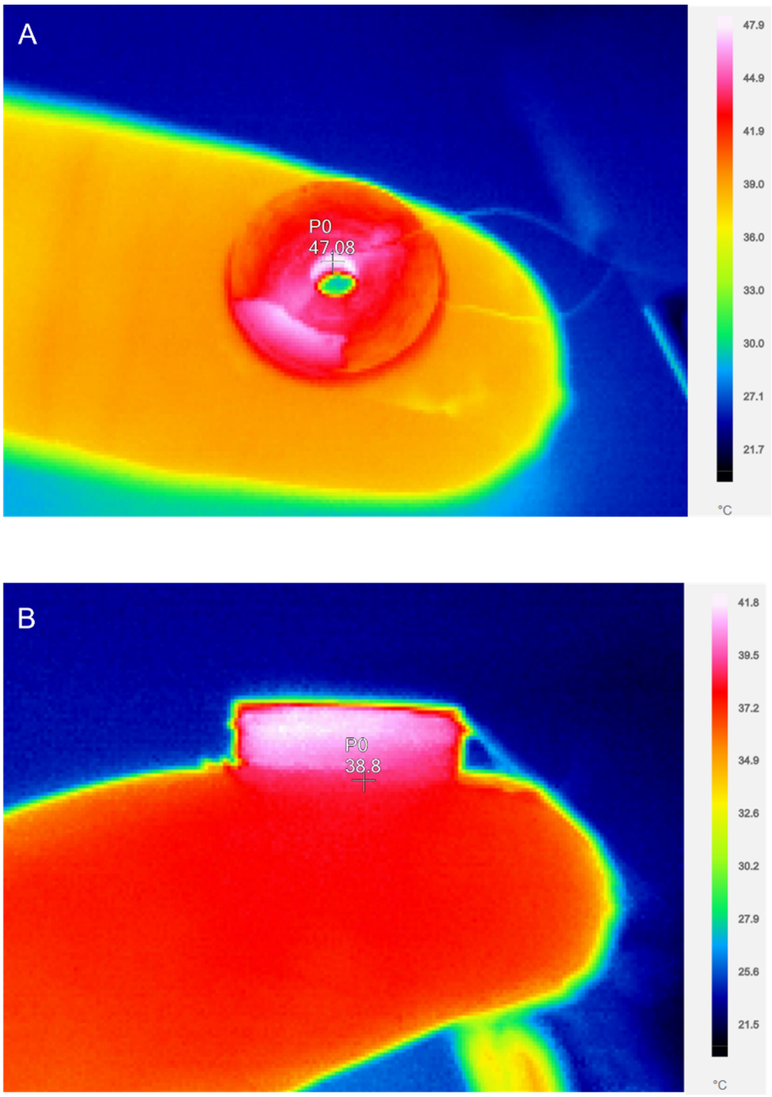

Fig. S14.

Infrared camera measurement of vibration actuator with highest feedback intensity. (A) Upper view **(B)** lateral view.

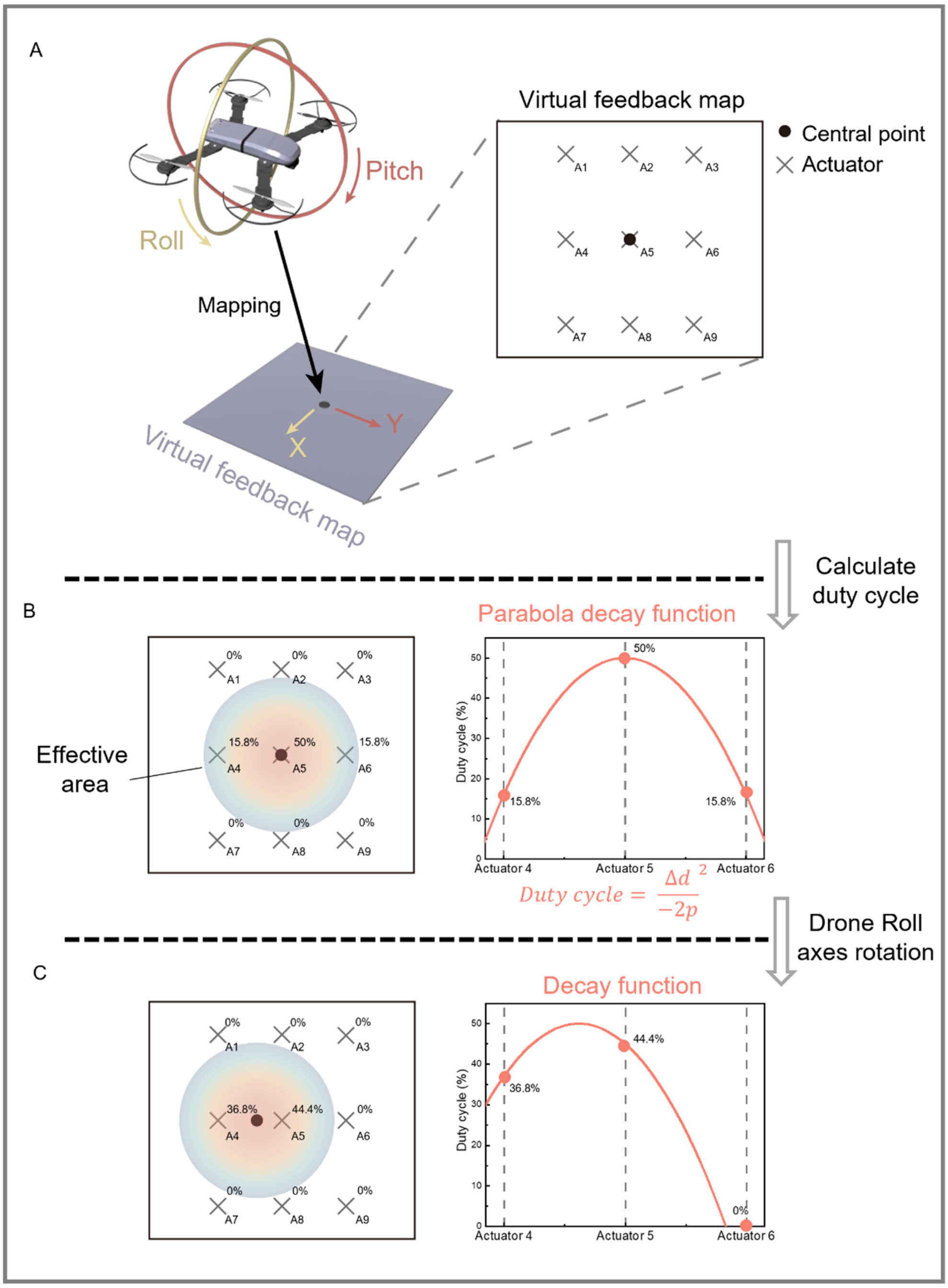

Fig. S15.

Mapping of Drone's angle in virtual tactile feedback map. (A) Conversion of the pitch and roll angle data of the drone into X, Y coordinates on the virtual feedback map, establishing the central point. **(B)** Calculation of the duty cycles of each actuator based on the decay function, forming the effective area. **(C)** Coordinated variation of the central point and effective area as the drone rotates, resulting in corresponding changes in the duty cycle of each actuator.

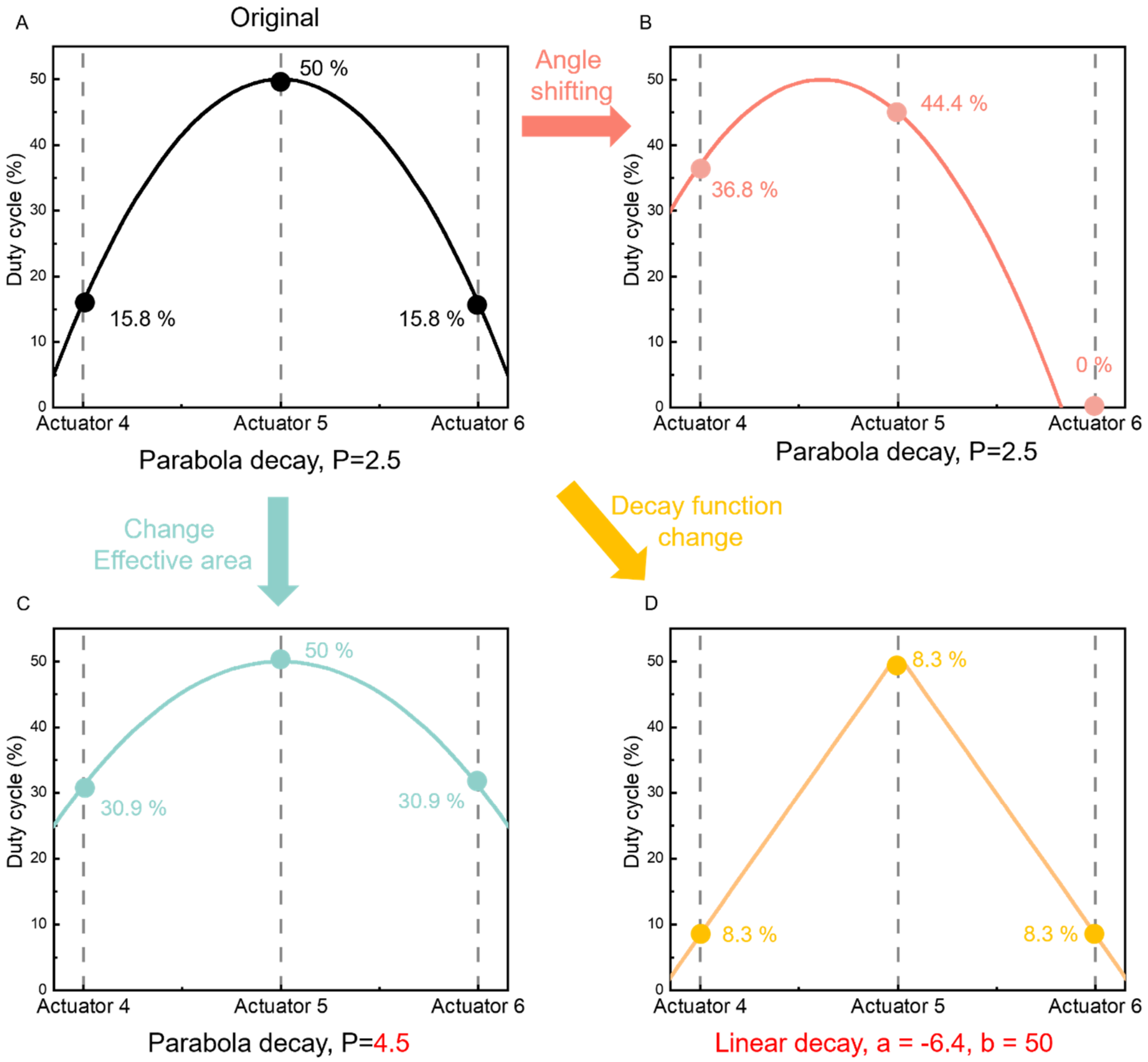

Fig. S16.

Variation in decay function. (A) Original parabola decay function with a focus of 2.5. **(B)** Decay function after rotation of the drone. **(C)** Decay function with a larger effective area, parabola decay function with a focus of 4.5. **(D)** Change of decay function to linear decay.

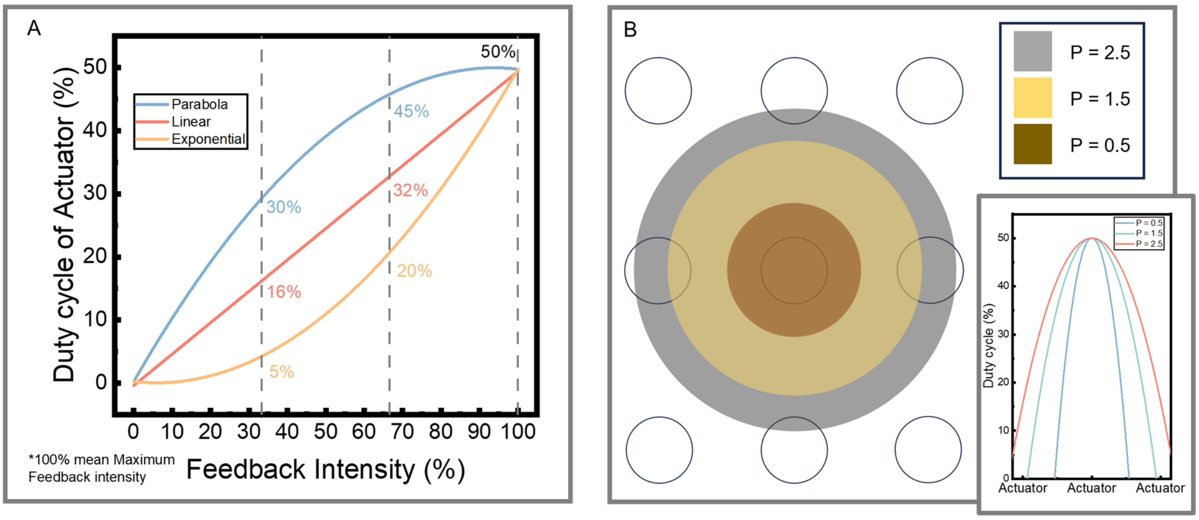

Fig. S17.

Different decay and effective area. (A) Three decay functions, parabola, linear, and natural exponential decay used in user study. **(B)** Effective areas utilized in the user study, established by parabolic decay functions with focuses of 0.5, 1.5, and 2.5.

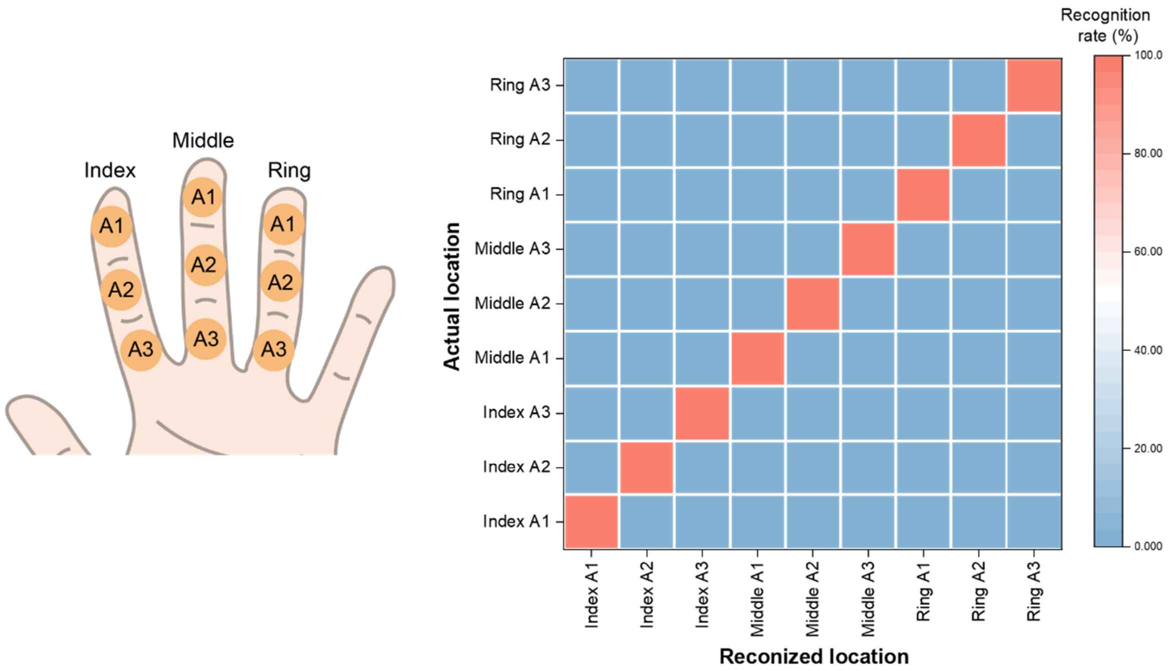

Fig. S18.

Assessment of mechanical crosstalk, recognition rate .

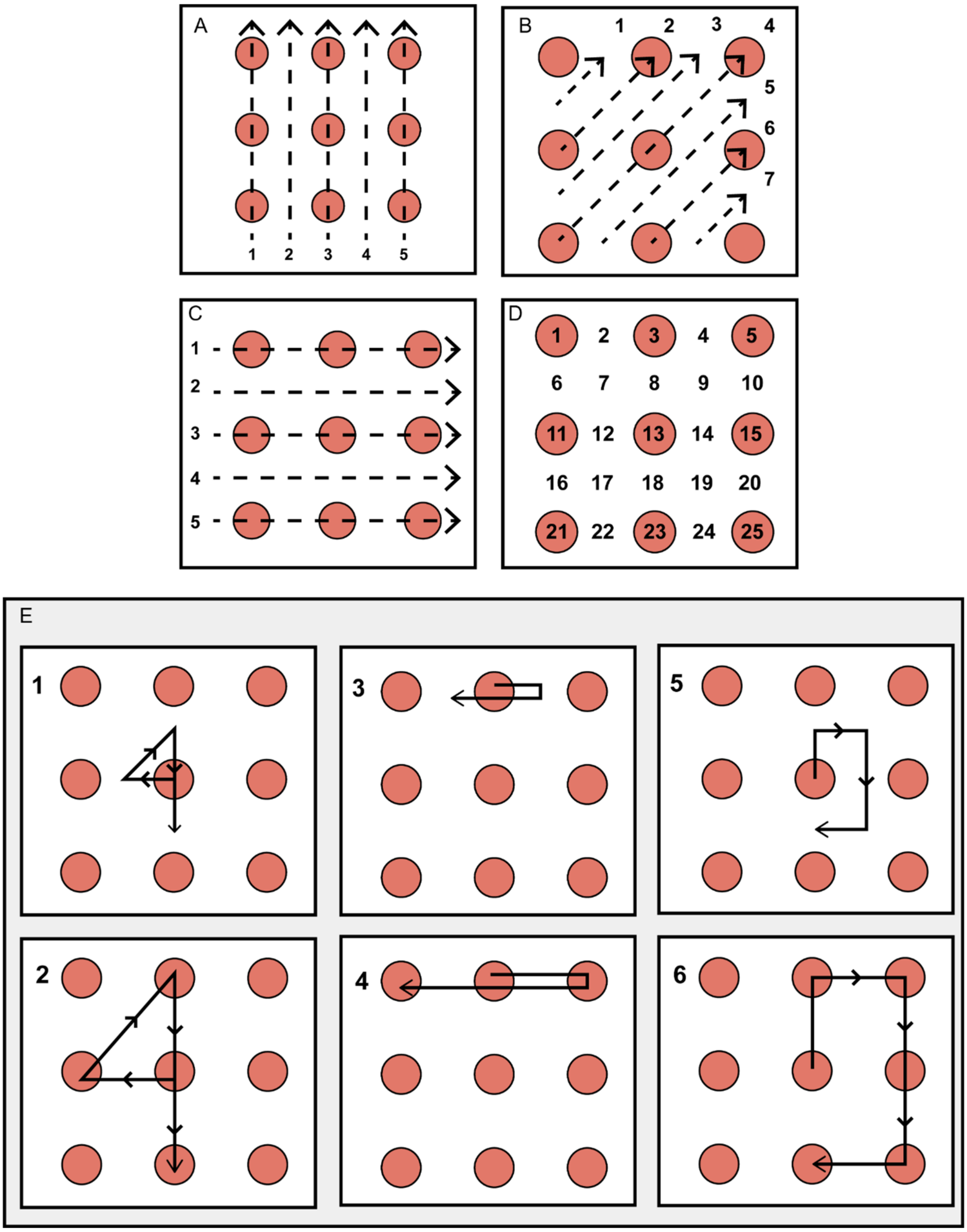

Fig. S19.

Pattern used in tactile feedback user study. (A) Five vertical sliding paths used in the study. **(B)** Seven oblique sliding paths used in the study. **(C)** Five horizontal sliding paths used in the study. **(D)** 25 locations used in single point test. **(E)** Six patterns used in complex sliding pattern test.

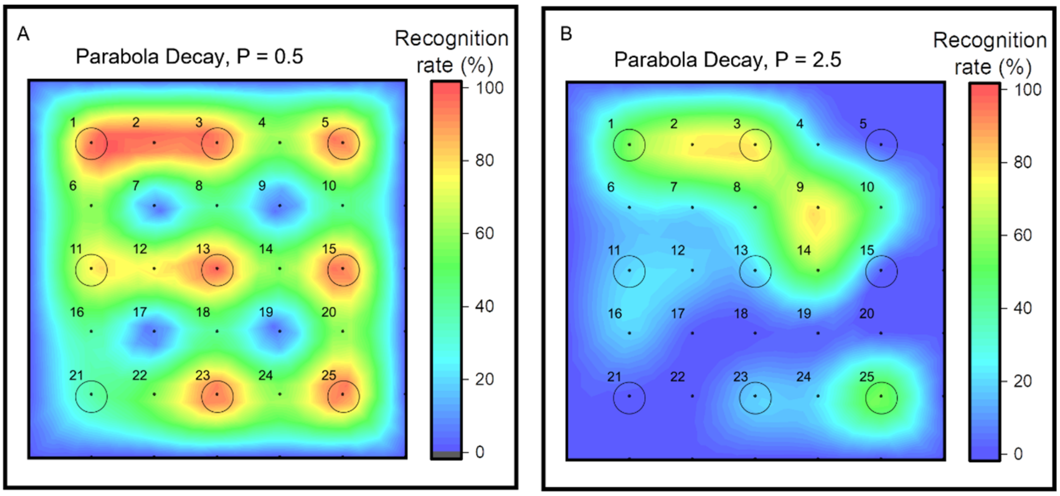

Fig. S20.

Result of single point test. (A) Average recognition rate of the single-point test when the effective area is similar in size to the parabolic decay with a focus of 0.5. **(B)** Average recognition rate of the single-point test when the effective area is similar in size to the parabolic decay with a focus of 2.5.

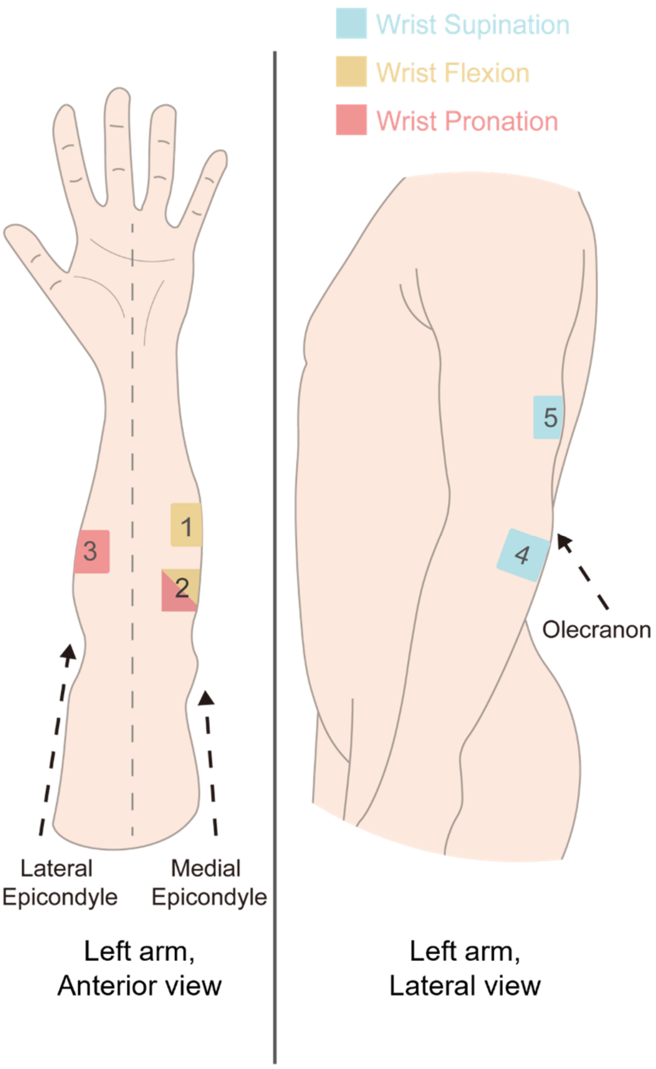

Fig. S21.

Electrode placement location for NMES force feedback.

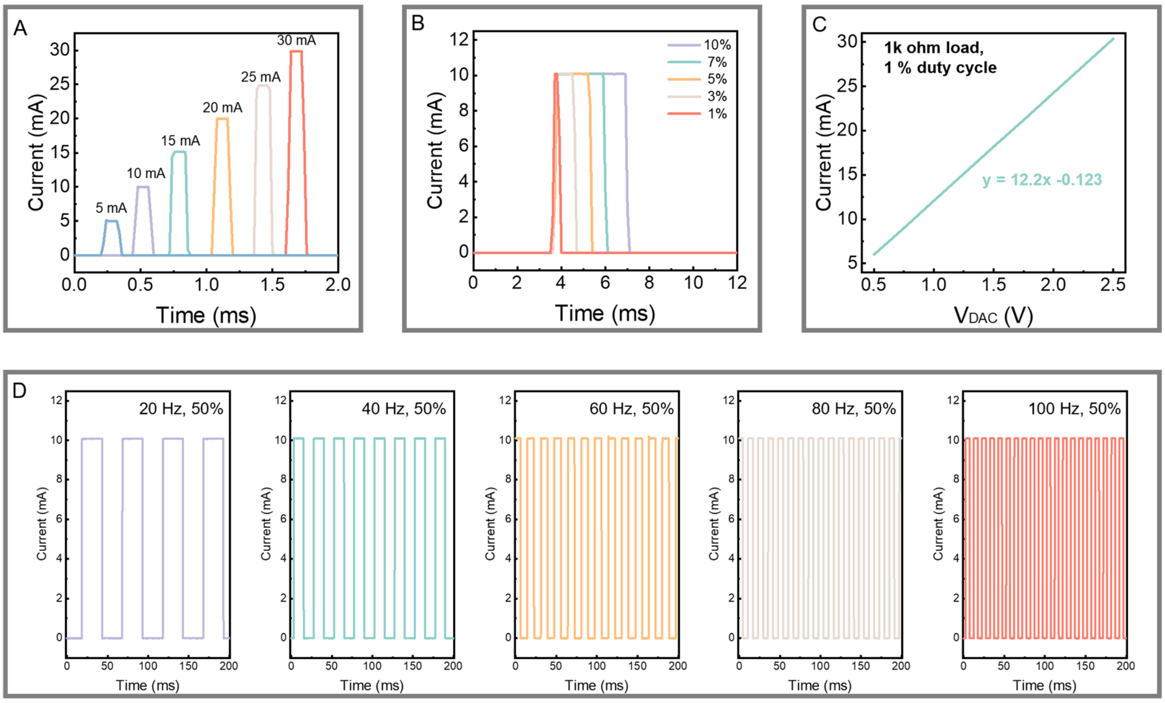

Fig. S22.

Electrical characteristic of stimulation output. (A) Variable current output from the NMESF modules. **(B)** Current pulse with different duty cycles output form NMESF modules. **(C)** The relationship between control voltage from DAC of NMESF control unit and the output current. **(D)** Variable frequency output from the NMESF modules.

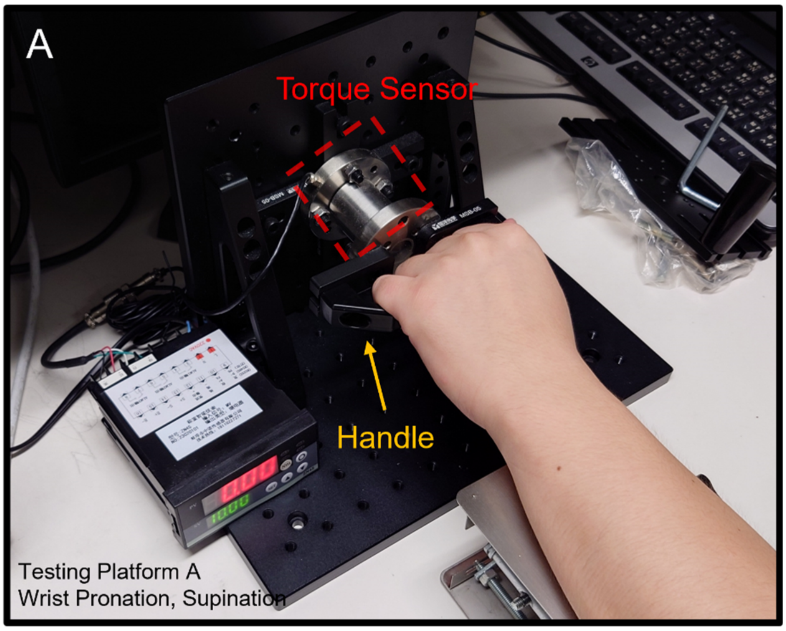

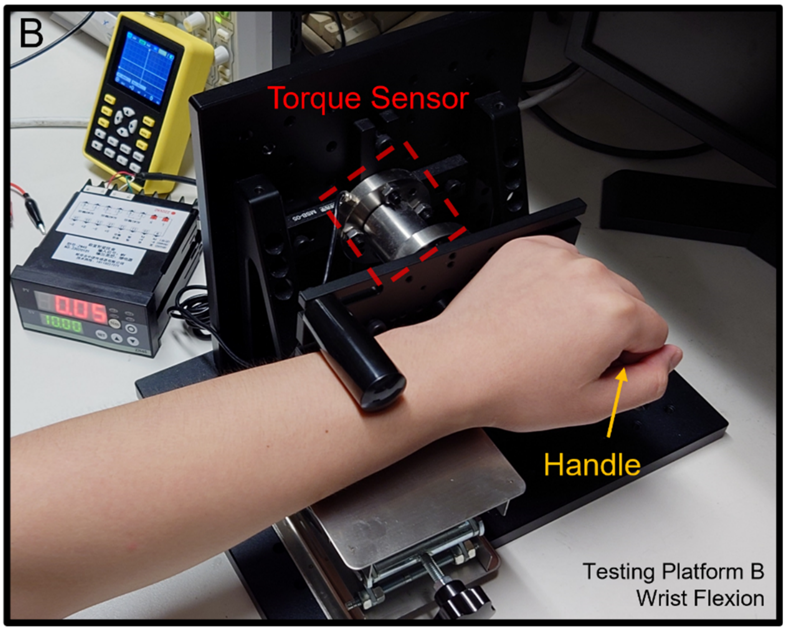

Fig. S23.

Torque testing platform. (A) Testing platform configurated for torque measurement of wrist pronation and wrist supination. **(B)** Testing platform configurated for torque measurement of wrist flexion.

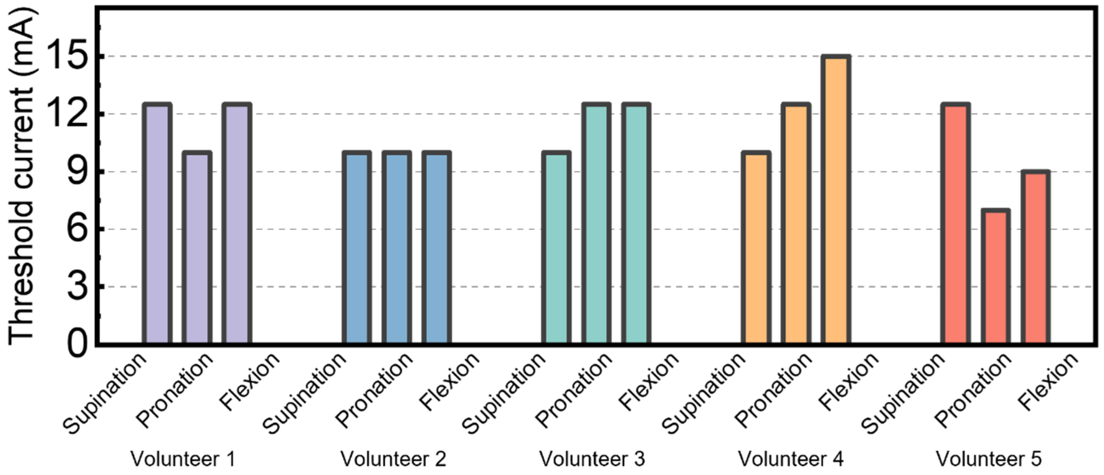

Fig. S24.
Motivation threshold of different volunteers.

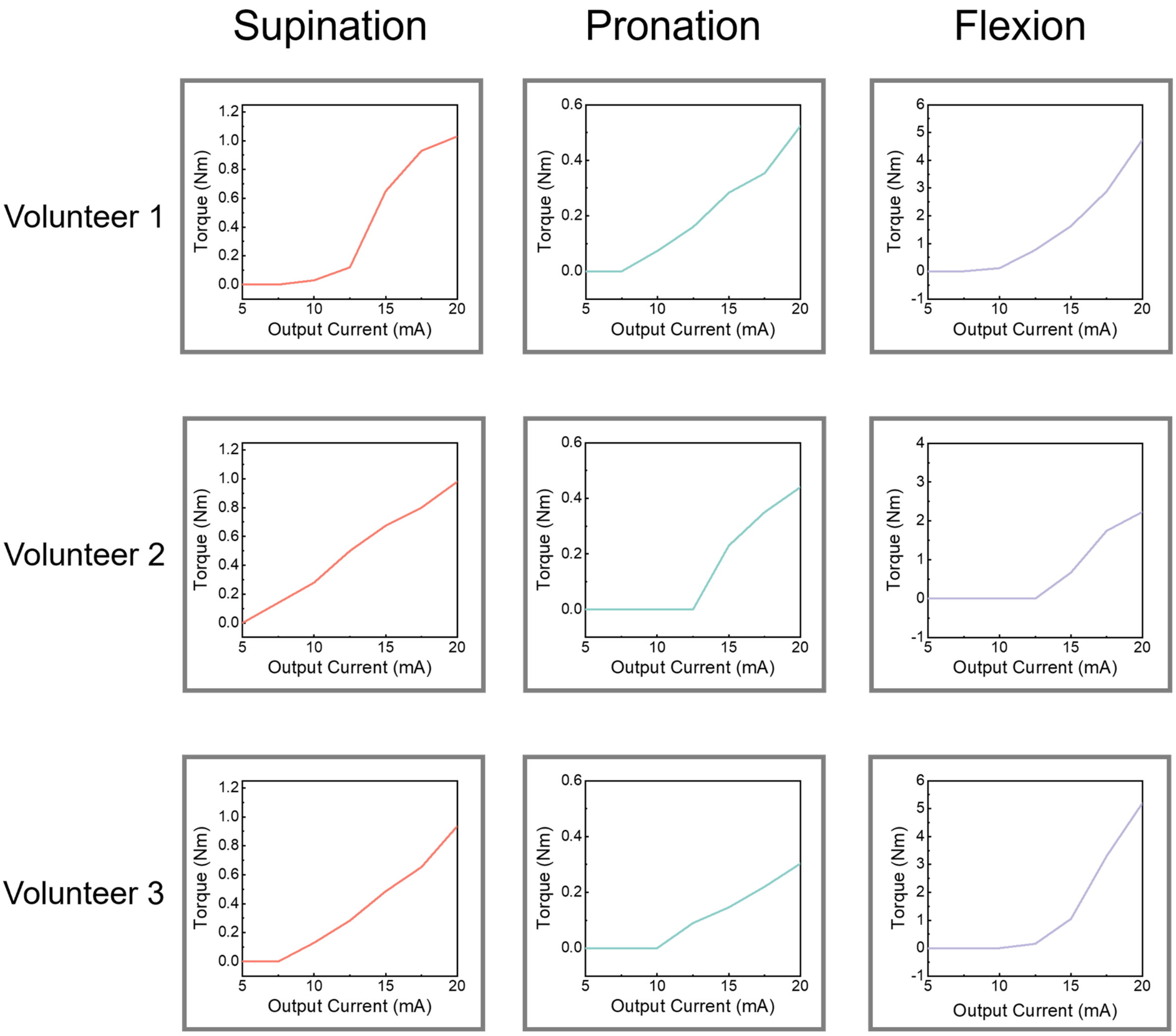

Fig. S25.

Raw torque data in three bending dimensions.

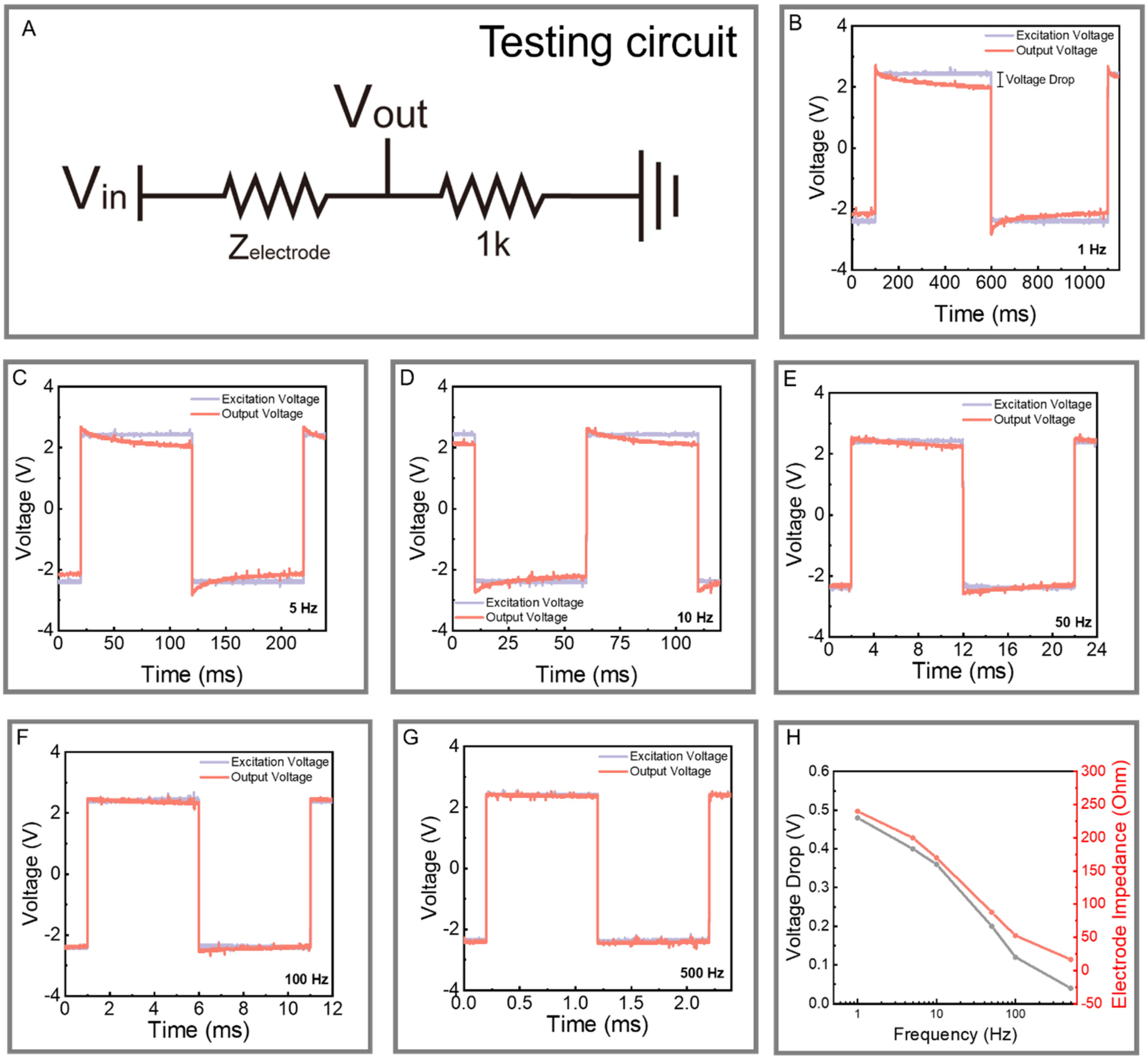

Fig. S26.

Frequency response of the stimulation electrode. (A) Testing circuit use for test the frequency response of the electrode. **(B)-(G)** Frequency response of the electrodes with excitation signal in different frequencies. **(H)** Relationship between frequency and impedance of electrode.

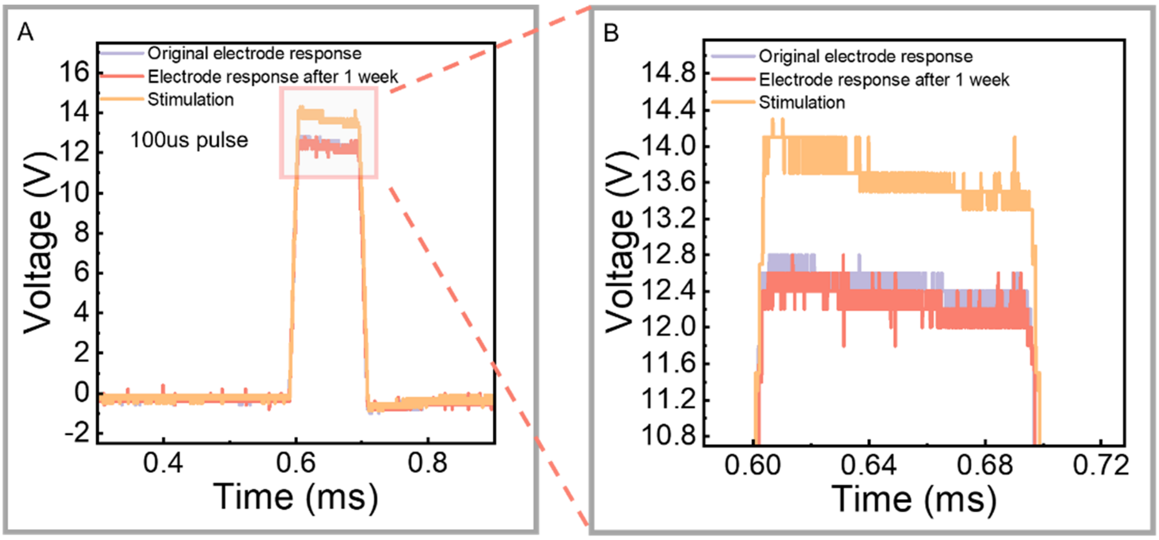

Fig. S27.

Long-term performance of the stimulation electrode. (A) The stimulation waveform (yellow line), the response of the new electrode (blue line), and the response of the electrode with hydrogel attachment for one week (red line). **(B)** Enlarged detail of panel A.

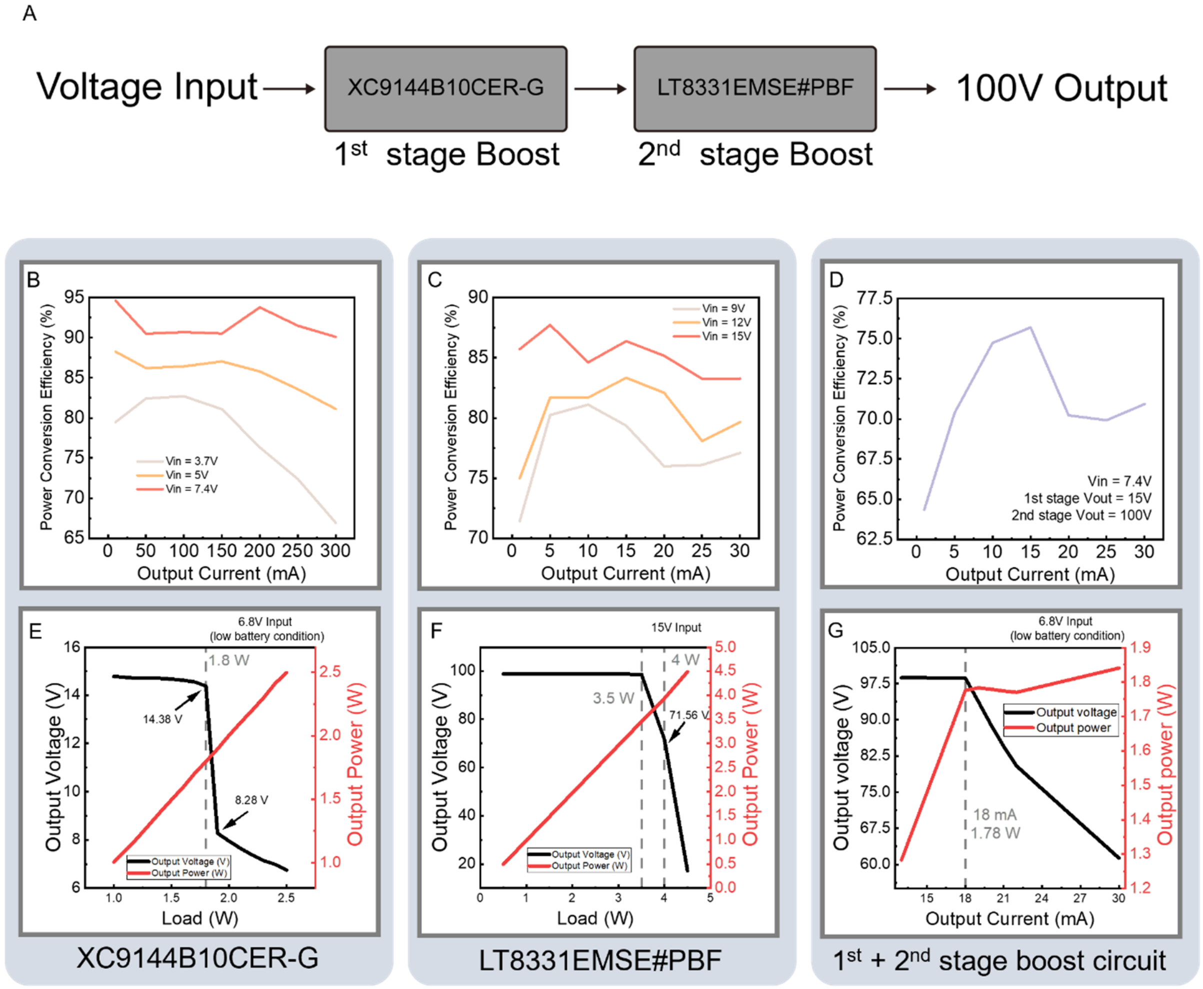

Fig. S28.

Output performance of the two-stage boost circuit. (A) Structure of the two stage boost circuit. **(B)** Power conversion efficiency of the first stage with different input voltage and output current. **(C)** Power conversion efficiency of the second stage with different input voltage and output current. **(D)** Power conversion efficiency of the two-stage boost circuit with optimized input voltage. **(E)** Relationship between output load and output voltage of the first stage. **(F)** Relationship between output load and output voltage of the second stage. **(G)** Relationship between output load and output voltage of the whole two-stage boost circuit.

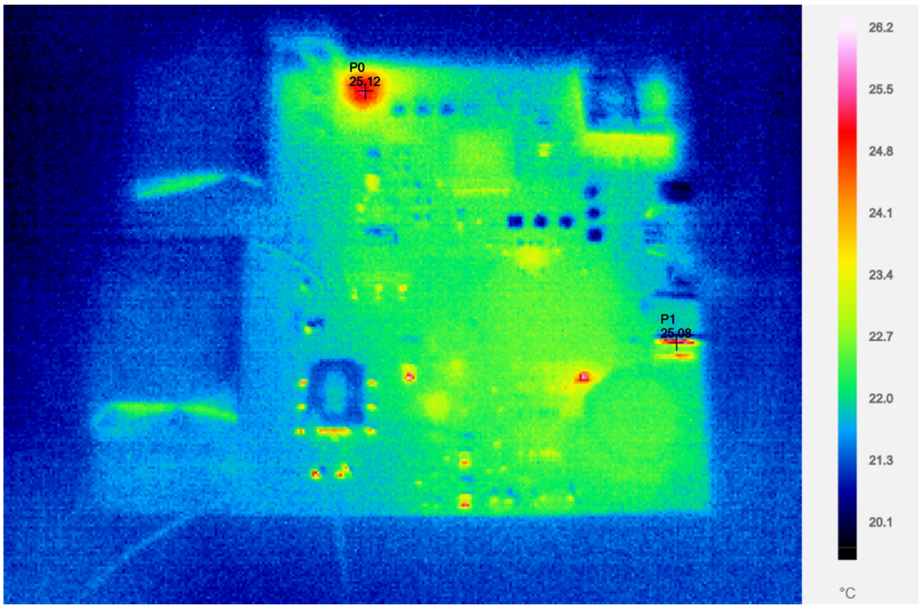

Fig. S29.
Infrared camera measurement of the NMESF circuit during operation.

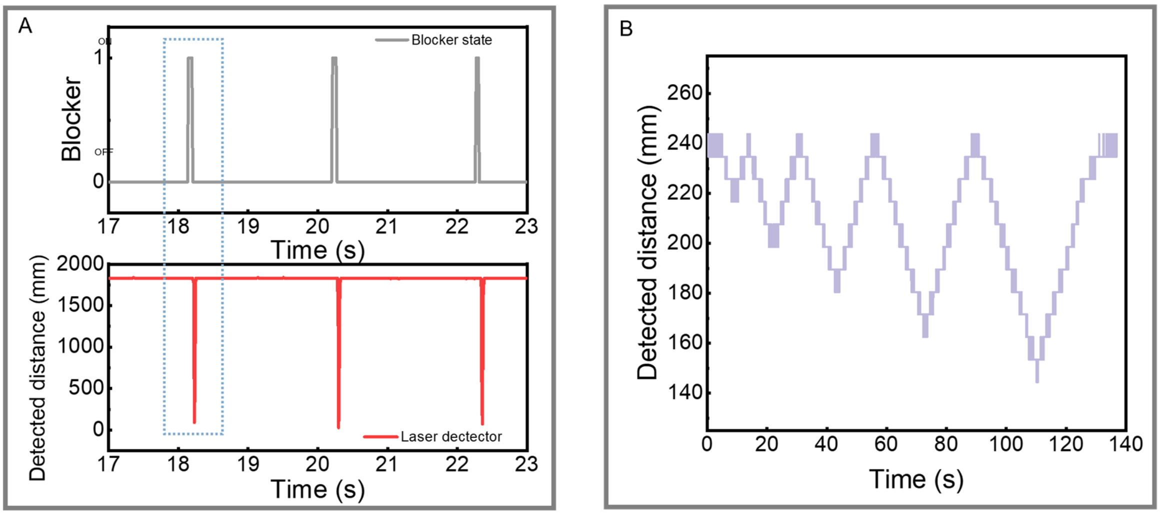

Fig. S30.

Evaluation of the obstacle detection system. (A) Response of the laser detector after detecting an obstacle, where the gray line indicates the presence of the obstacle and the red line represents the reading of the laser detector. **(B)** Accuracy test of the laser detector.

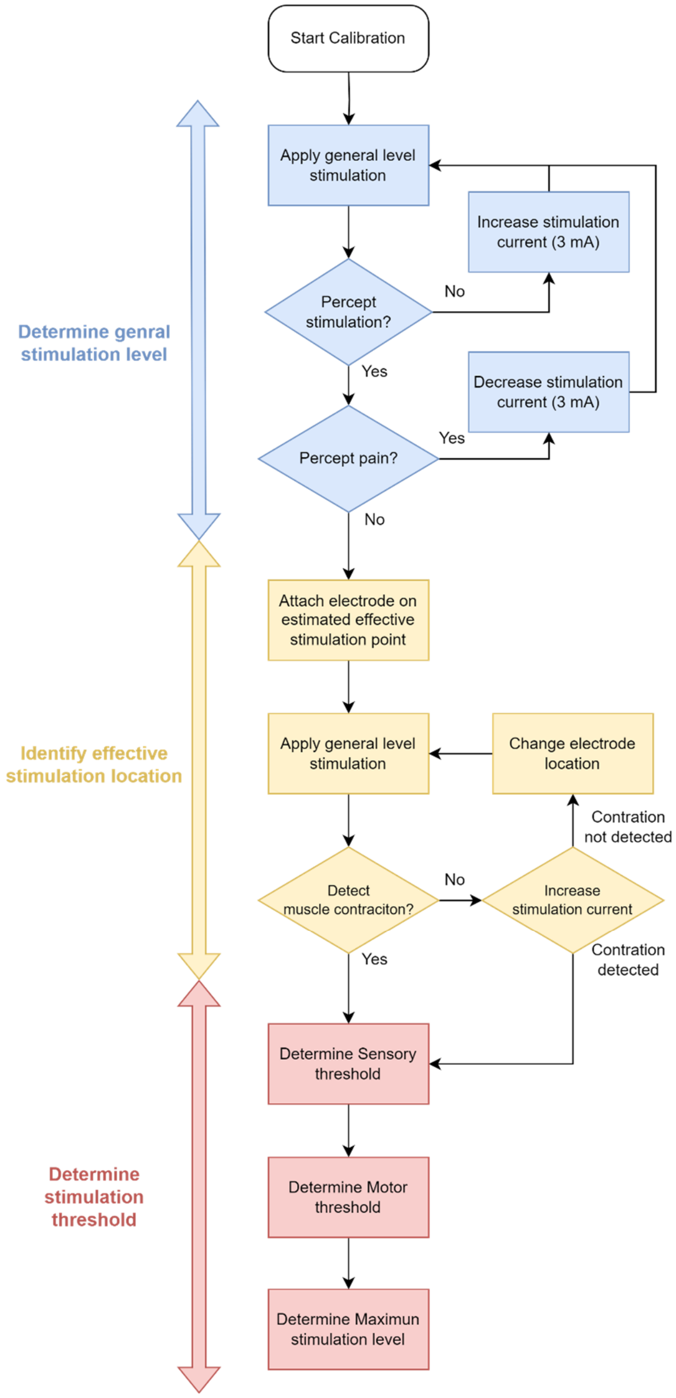

Fig. S31.

Calibration procedure of the NMES force feedback system.

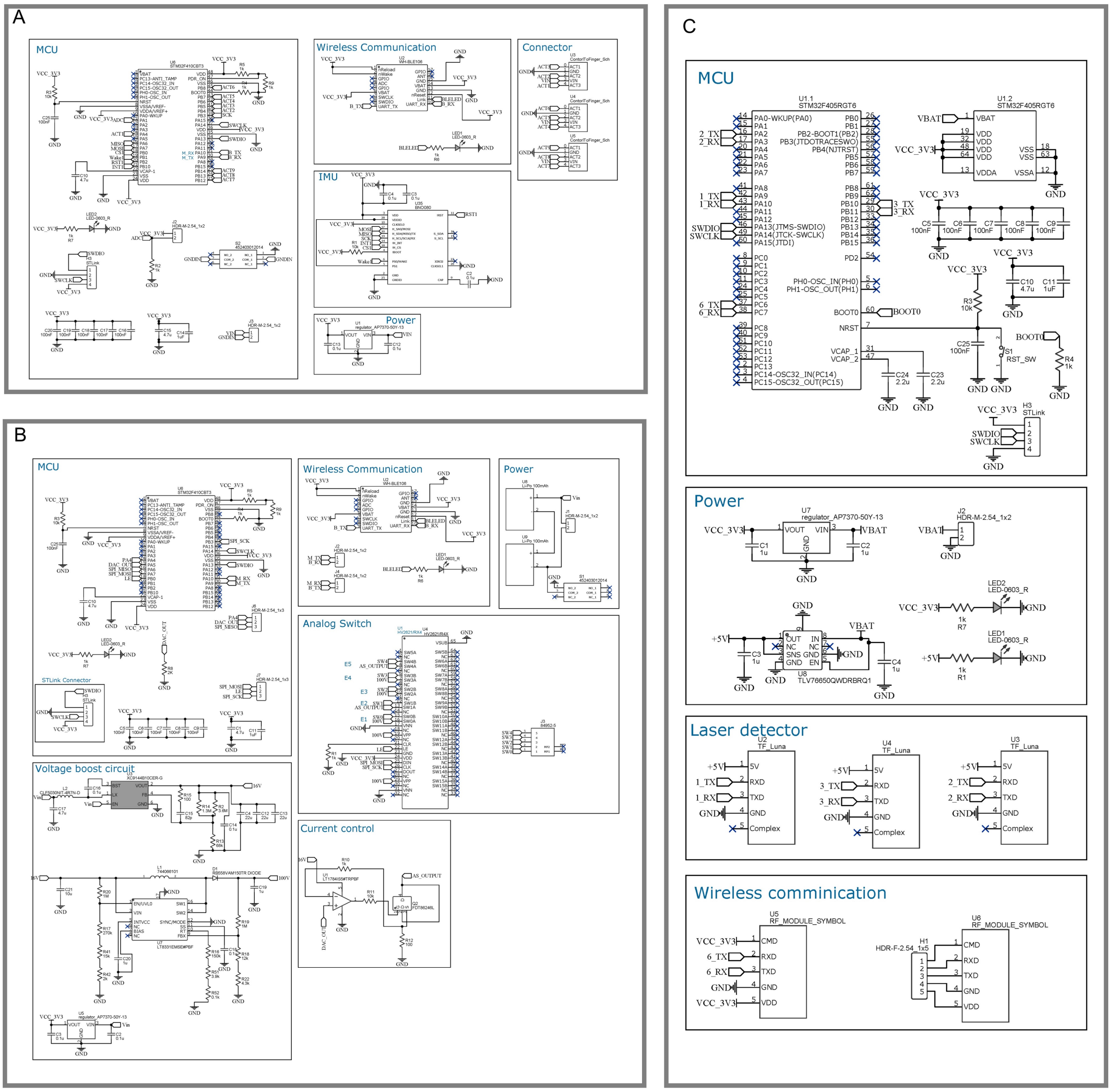

Fig. S32.

Detail circuit design for the 3 major functionality modules. (A) DCTF modules. **(B)** NMESF module. **(C)** Obstacle detection system.

Movie S1.
Vibration amplitude of the haptic actuator under different frequencies.

Movie S2.
Vibration amplitude of the haptic actuator under different duty cycles.

Movie S3.
Demonstration of the system avoiding collision of stationary obstacle behind the drone.

Movie S4.
Demonstration of the system avoiding collision of moving obstacle beside the drone.
